# Interactions of tropomyosin Tpm1.1 on a single actin filament: A method for extraction and processing of high resolution TIRF microscopy data

**DOI:** 10.1371/journal.pone.0208586

**Published:** 2018-12-10

**Authors:** Miro Janco, Till Böcking, Stanley He, Adelle C. F. Coster

**Affiliations:** 1 Single Molecule Science and ARC Centre of Excellence in Advanced Molecular Imaging, University of New South Wales, Sydney, New South Wales, Australia; 2 School of Mathematics & Statistics, University of New South Wales, Sydney, New South Wales, Australia; University of Bonn, GERMANY

## Abstract

Skeletal muscle tropomyosin (Tpm1.1) is an elongated, rod-shaped, alpha-helical coiled-coil protein that forms continuous head-to-tail polymers along both sides of the actin filament. In this study we use single molecule fluorescence TIRF microscopy combined with a microfluidic device and fluorescently labelled proteins to measure Tpm1.1 association to and dissociation from single actin filaments. Our experimental setup allows us to clearly resolve Tpm1.1 interactions on both sides of the filaments. Here we provide a semi-automated method for the extraction and quantification of kymograph data for individual actin filaments bound at different Tpm1.1 concentrations. We determine boundaries on the kymograph on each side of the actin filament, based on intensity thresholding, performing fine manual editing of the boundaries (if needed) and extracting user defined kinetic properties of the system. Using our analytical tools we can determine (i) nucleation point(s) and rates, (ii) elongation rates of Tpm1.1, (iii) identify meeting points after the saturation of filament, and when dissociation occurs, (iv) initiation point(s), (v) the final dissociation point(s), as well as (vi) dissociation rates.

All of these measurements can be extracted from both sides of the filament, allowing for the determination of possible differences in behaviour on the two sides of the filament, and across concentrations. The robust and repeatable nature of the method allows quantitative, semi-automated analyses to be made of large studies of acto-tropomyosin interactions, as well as for other actin binding proteins or filamentous structures, opening the way for dissection of the dynamics underlying these interactions.

## Introduction

Direct observation of interactions between single molecules and various filamentous structures (including DNA, microtubules, intermediate and actin filaments) using fluorescence microscopy is quickly becoming a new standard in many fields of life sciences [1–4]. An important aspect of such assays is not only specific experimental design and data acquisition, but also sensible analysis of acquired data. To date, there have been several very useful analytical tools published for the resolution, processing and interpretation of fluorescence-based binding kinetics for different types of cellular filaments [5–11]. These tools are designed for a wide range of experimental designs, and are able to resolve data at low signal-to-noise ratio in cases of microtubule end-tracking [6], actin filament tracking [11] and complex biopolymer 3D networks [10]. Single particle tracking algorithms such as FIESTA are able to localize centreline of curved microtubule filaments with 2 nm precision and at the filament tip with 9 nm precision by fitting a two-dimensional model to fluorescently-labelled microtubules [7]. There are also more generic analytical packages available, such as ImageJ, for a quick manual analysis of filaments, and automated packages for tracking and quantification of kymographs [12]. The selection of analytical packages described above provides rapid and precise detection and extraction of fluorescence traces along various filamentous structures, often followed by formation of kymographs for spatio-temporal analysis. Studies targeting proteins, which bind to the actin filament in multiple layers (tropomyosin, coffilin), or form actin filament bundles (villin, fimbrin and LIM proteins), often produce kymographs with distinct layers of intensity, formed by the overlapping stretches of labelled proteins of interest [1, 13, 14]. The appearance of distinct intensity regions, corresponding to different physical distributions, indicates the need to develop robust and quantitative methods with the potential to recognise and extract regions of distinct intensities on the filament–which is the subject of our study.

Tropomyosins belong to the group of proteins, which act as major regulators of the interactions between filamentous actin and other actin binding proteins. There are over 40 mammalian tropomyosin isoforms involved in the regulation of actin filament functions in time and space. Among such a variety of isoforms, the striated muscle tropomyosin Tpm1.1 has undoubtedly been the most studied tropomyosin isoform over the last 50 years. It is an alpha helical coiled-coil dimer that forms a polymer along each of the major grooves of the actin filament and is involved in the calcium-dependent regulation of muscle contraction [15, 16]. Whilst theories exist regarding the mechanism of association and dissociation of tropomyosin isoforms with actin, experimental evidence for tropomyosin interactions with actin filament at single filament/molecule level has recently been extended by several significant studies [1, 17–19].

Recently, we used total internal florescence (TIRF) microscopy to investigate the effects of surface chemistry on the binding kinetics of the protein tropomyosin (isoform Tpm1.1) to individual actin filaments in an *in vitro* reconstitution assay [11]. Our data showed two distinct intensities of AlexaFluor-647 labelled Tpm1.1, bound to actin filaments. We attributed these two levels of intensity to the stretches of actin filament decorated by Tpm1.1 on one or both sides of the filament. Here we describe in detail the methodology for processing and analysis of our data.

**Fig 1:**
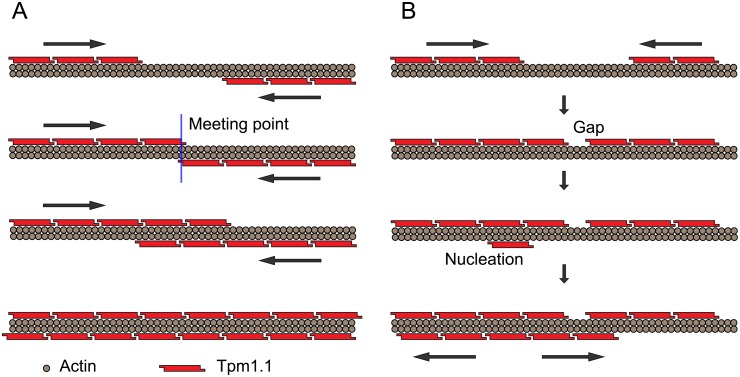
Schematic of Tpm1.1 binding to both sides of actin filament. (A) Growth of Tpm1.1 strands on both sides on actin filament in opposite directions. The meeting point where two intensities of fluorescently labelled Tpm1.1 start to overlap is indicated with the blue line. This type of Tpm1.1 association with actin leads to fully saturated filament. (B) Initial growth of Tpm1.1 strands on the same side of actin can lead to a gap formation or the fully saturated region. A single Tpm1.1 isomer stretches over seven actin monomers; therefore gaps may be formed by short stretches of undecorated actin filament with a length of 1 to 6 actin monomers.

When Tpm1.1 binds to “naked” actin filaments, a single Tpm1.1 isomer initially associates with a section of seven actin monomers on one side of an actin filament–nucleation. Other Tpm1.1 monomers subsequently bind next to this bound Tpm1.1 monomer, growing a Tpm1.1 strand. More nucleations to unbound regions and the growth of the strands can occur on either side of the actin filament. A schematic of the processes and configurations of Tpm1.1 binding to an actin filament is shown in Fig 1.

Using single molecule fluorescence microscopy combined with microfluidics techniques, we show the measurement of Tpm1.1 binding to single actin filaments and subsequent dissociation upon removal of Tpm1.1 from the surrounding solution. Analyses are then developed to derive the dynamics of the processes and other information from the image sequences obtained experimentally, and the inherent limitations of experimental resolution are determined.

## Experimental materials and methods

### TIRF and microfluidic setup

Actin-tropomyosin interactions were observed by using total internal reflection fluorescence (TIRF) inverted microscope (TILL Photonics) equipped with a Zeiss 100× Plan-apochromat NA 1.46 oil objective and Andor iXon 897U EMCCD cameras. Lasers used were 488 nm and 640 nm (Toptica photonics AG) with 525/50-25 and 697/75-25-D single-band filters, respectively (BrightLine, Semrock). Time-lapse images were acquired typically at 10 Hz for Tpm association and 1 Hz for dissociation. The laser power was 0.44 mW and 0.30 mW for the 488 nm and 640 nm lasers, respectively. The exposure time was 20 ms and the multiplication gain was 300 for both channels.

#### Protein expression, purification and labelling

Rabbit skeletal actin in its globular form was purchased from Hypermol EK, Germany (Cat. #: 8101–01). G-actin (250μg) was resuspended in 250 μl of H_2_O to obtain a stock solution of 1 mg/ml. Stock solution was left to rehydrate at room temperature for 2 min and then dissolved using pipette.

Murine tropomyosin isoform Tpm1.1 was expressed in BL21 (DE3)-RP and purified as described previously [20] with some modifications. Tpm1.1 was expressed in 2 L of LB broth containing 100 μg/ml ampicillin at 37°C. When cell density reached OD_600_ 0.6, the expression was induced by addition of isopropylthio-b-D-galactosidase (IPTG, Gold Biotechnology) at 1mM. Cells were incubated for additional 3 hours and harvested by centrifugation (8,280 g, 4°C for 15 min, SLA-3000 rotor, Thermo Scientific). The pellet was resuspended in lysis buffer (20 mM sodium phosphate pH 7.5, 500 mM NaCl, 5 mM MgCl_2_, 1 mM NaN_3_, 2 μg/ml of DNAse I (Sigma Aldrich), 1 mM phenylmethanesulfonyl fluoride (PMSF, Sigma Aldrich) and “Complete” protease inhibitor cocktail tablet (Roche)). Cells were sonicated (30 s pulse on/30 s pulse off cycle, repeated 4 times on ice) and then immediately heated to 80°C for 10 min. The lysate was cooled to room temperature and centrifuged at 47,810 g, 4°C for 45 min (SS-34 rotor, Sorvall RC6C, Thermo Scientific) to remove cell debris. The supernatant pH was dropped to 4.7 and precipitated material was centrifuged at 3900 g, 4°C for 12 min (SX 4250 rotor, Alegra X-22 R, Beckman Coulter). The pellet was resuspended in buffer A (150 mM NaCl, 10mM Tris pH 7.5, 2 mM DTT, 1 mM NaN_3_, 0.5 mM EDTA) and the acidification procedure was repeated two more times. Protein was then loaded onto a 2x5 ml HiTrap HP anion exchange columns (GE Healthcare) equilibrated with buffer A and washed with 10 CV of buffer A. Tpm1.1 was then eluted by linear gradient from 150–1000 mM NaCl over 10 CV, protein peak fractions were analysed by SDS-PAGE, pooled and concentrated by Amicon Ultra-15 centrifugal filters (Merc Millipore). Tpm1.1 was then loaded onto HiLoad 16/600 Superdex 200 pg (GE Healthcare) size exclusion chromatography column equilibrated with SEC buffer (150 mM KCl, 5 mM Tris pH 7.8, 2 mM DTT, 1 mM MgCl_2_, 1 mM NaN_3_, 0.2 mM EGTA, 0.1 mM CaCl_2_) over 1 CV. Protein fractions were again analysed by SDS-PAGE, pooled, concentrated, snap-frozen, lyophilised and stored at -30 °C. Additionally, the alanine-serine extension was inserted to the N-terminus to mimic the N-acetylation of the native tropomyosin [21]. Tpm1.1 was labelled with Alexa Fluor 647 C_5_-maleimide (Life technologies) at Cys-190 and the labelling ratio of 20% as described in Nicovich, et al. [11]. Lyophilized Tpm1.1 was resuspended in 900 μl of the labelling buffer (PBS 1x pH 7.4, 300 mM NaCl) to a final concentration 10mg/ml. 20 mM of DTT was then added and the sample was transferred into the heating block for 2h at 38°C to ensure that Tpm1.1 is in monomeric form. Reducing agent (DTT) was then removed using Zeba Spin desalting columns (7k MWCO, 2ml, Thermo Fisher scientific). Alexa Fluor 647 C_5_-maleimide was then added to the protein sample in 5:1 ratio, respectively and incubated at RT for 1 hour. Excess dye was removed using Zeba Spin desalting columns and the labelling ratio was determined by UV-visible light absorption spectroscopy. Single molecule photobleaching of labelled Tpm1.1 showed majority (81%) of single-step bleaching traces indicating predominant population of singly labelled Tpm1.1 dimers. Effect of labelling on affinity for the actin filament was tested by titrations of labelled Tpm1.1 mixed with increasing concentration of unlabelled Tpm1.1 up to molar ratio of 1:14. At the molar ratio of 1:7 (labelled:unlabelled), AF647-Tpm1.1 showed robust staining along the actin filament and further decrease of labelled Tpm1.1 to 1:14 molar ratio exhibited a punctuate staining pattern. The presence of the labelled Tpm1.1 on the filament at high excess of unlabelled Tpm1.1 indicated that labelling of of Tpm1.1 at Cys-190 has no significant effect on affinity to filamentous actin.

Spectrin seeds were prepared by the method of Casella et al., [22] and biotinylated by Sulfo ChromaLink biotin (Solulink). Spectrin (260 μl of 0.2 mg/ml protein stock in M buffer containing 100mM sodium phosphate pH 8, 150 mM NaCl) was mixed with 0.5 mg of biotin dissolved prior to the reaction initiation with 100 μl of dH_2_O. Mixture was incubated at RT for 120 min and excess of biotin was then removed using Zeba Spin desalting columns. Degree of biotinilation was measured by UV-spectroscopy and E1% ChromaLink biotin MSR calculator (Solulink), indicating 3 biotins per spectrin molecule.

#### Formation of the actin-tropomyosin polymer in the microfluidic channel

Microfluidic devices were prepared according to protocol from Böcking et al., [23], described in detail below. Flow cells and modification of the coverslip surface were prepared as described in Nicovich, et al. [11]. The microfluidic device was assembled by attaching a cured polydimethylsiloxane (PDMS) five channel cube to the coverslip (Marienfeld superior No. 1.5H, 24 mm x 60 mm). Prior to attachment, the coverslip was sonicated in 100% ethanol and 1 M NaOH followed by a thorough dH_2_O rinse between these steps. The PDMS cube was washed with isopropanol and dried with N_2_ gas. The PDMS cube and the coverslip were then exposed to an air plasma (700 torr) for 3 min. The PDMS cube was attached to the coverslip to form the final microfluidics device. The channels of the device were filled with a copolymer of poly-L-lysine (PLL) and biotinylated poly(ethylene glycol) (PEG) [Susos AG, PLL(20)-g[3.4]-PEG(2)/PEG(3.4)-biotin (20%)] in PBS (7 μL, 1 mg mL^-1^) and incubated for 20 min. The microfluidic device was at this stage connected to a syringe pump via tubing, and the excess of PLL-PEG was washed out with 95 μL of PBS. The channel was then loaded with 1% pluronic F127 (Sigma-Aldrich) and 50 μg mL^-1^ kappa casein solution in PBS, incubated for 1 min and washed with 95 μL of PBS. The next step was a streptavidin (Thermo Fisher Scientific) load (15 μL at 1mg mL^-1^ and 10 min incubation) with subsequent wash with 95 μL of buffer G (5 mM Tris pH 7.8, 1 mM 1,4-Diazabicyclo[2.2.2]octane (DABCO, Sigma Aldrich), 0.2 mM ATP (Sigma Alrdich, Grade I, ≥99%), 0.1 mM CaCl_2_, 0.01% NaN_3_, 10 mM DTT, 1 mg mL^-1^ BSA). Biotinylated spectrin-actin seeds were then added (20 μL at 0.04 mg mL^-1^), incubated for 10 min and washed with 95 μL of buffer F (5 mM Tris pH 7.8, 100 mM KCl, 1 mM DABCO, 0.2 mM ATP, 1 mM MgCl_2_, 0.2 mM EGTA, 0.1 mM CaCl_2_, 0.01% NaN_3_, 10 mM DTT, 1 mg mL^-1^ BSA). The channel was at this point ready for actin polymerization, which was triggered by addition of 100 mM KCl into 1 μM of G-actin (final concentration). The activated actin solution (20 μL) was then loaded into the channel and incubated for 3 min. Filaments were at this stage tethered to the coverslip at just one point and the rest of the filament is stretched above the coverslip surface, allowing Tpm1.1 to access all actin binding sites without any obstructions. Actin filaments were then visualized by staining with a 0.15 μM solution of Alexa Fluor 488-phalloidin (ThermoFisher Scientific), and ready for Tpm1.1 loading at various concentrations.

## Results

### Registering image stacks acquired for Tpm1.1 association and for subsequent dissociation experiments

**Fig 2:**
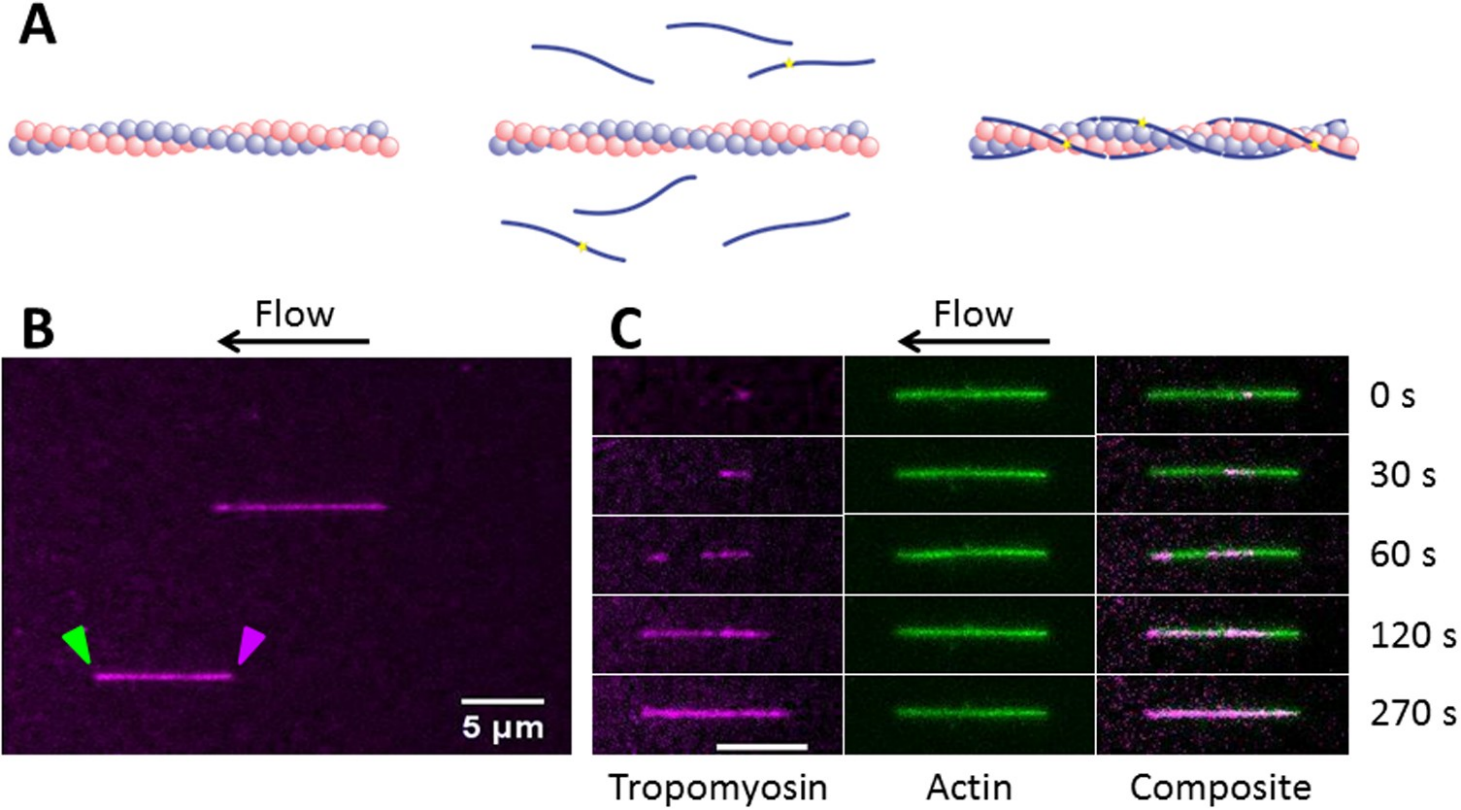
Association of tropomyosin Tpm1.1 to actin filaments. (A) Schematic of Tpm1.1 molecules binding to actin filament. (B) Actin filaments decorated with AF647-Tpm1.1 under the flow in the microfluidic channel. Green and magenta arrowheads indicate barbed end and pointed end of the actin filament, respectively. (C) Snapshots of a single actin filament from a two colour TIRF time lapse image stack show binding of Tpm1.1 to phalloidin-AF488 labelled pre-formed actin filament. Data shown in Fig 2B and C were for Tpm1.1 at 62.5nM at 92nm/pixel resolution and 1Hz acquisition speed. Scale bar, 5 μm.

TIRF microscopy image stacks were obtained during the process of Tpm1.1 isomer association with actin filaments. The schematic of Tpm1.1 association and typical images obtained are shown in Fig 2. Once the actin filaments were formed, association was initiated by flowing Tpm1.1 into the microfluidic channel. As soon as association reached the saturation point, Tpm1.1 was then removed from the flow system, and dissociation was observed from the actin filament.

The image stacks in the association phase at Tpm1.1 concentrations above 62.5nM were typically acquired at 10 Hz, due to faster binding kinetics of reaction. The dissociation phase occurred at a slower rate and was recorded separately at 1Hz. The association and dissociation image stacks were aligned using two-dimensional intensity-based image registration of a late stage association phase image and an early stage dissociation image. The images were assumed to not be distorted, so a rigid transformation of translation and rotation was optimised using regular step gradient descent.

### Extraction of individual filament kymographs

Software for filament tracing and extracting filament dynamics from fluorescence images was developed [11], to trace filament backbones in fluorescence images. Using this software, a kymograph of the intensity of the filament along the backbone versus time can be obtained. In the case of straight filaments shown in our study, other software such as ImageJ can also be used to extract the kymographs–calculating the average intensity down a straight path in the image.

Kymographs were extracted using the registered image stacks of both the association and dissociation phases of the Tpm1.1 isomers with actin filaments. Usually multiple filaments were in the field of view, so multiple kymographs of the association and dissociation of filaments could be extracted.

**Fig 3:**
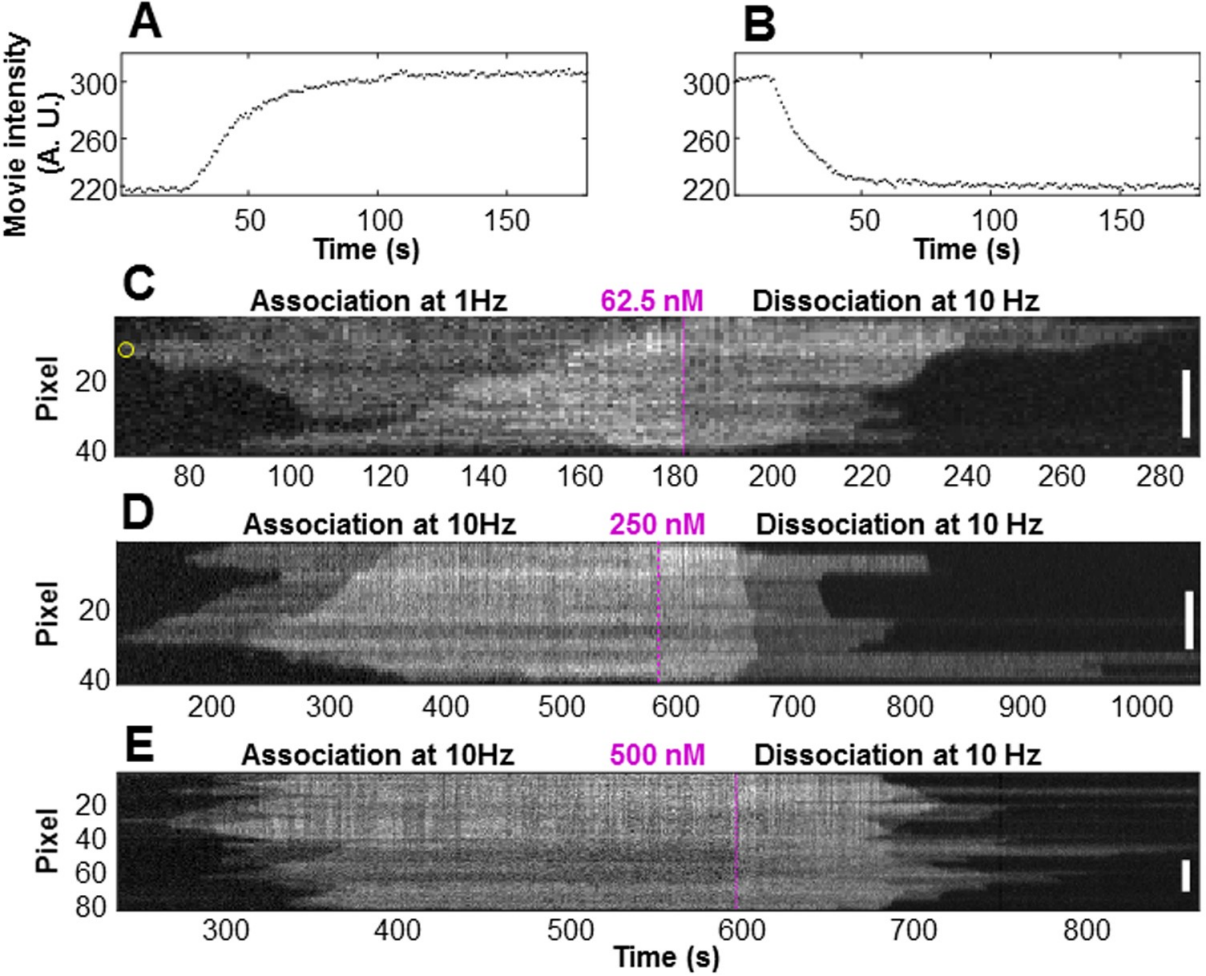
Intensity profile and kymographs of tropomyosin Tpm1.1 at different concentrations. (A) The total intensity of the region of interest (ROI) as a function of time during the association phase. The ROI is selected by the user in the image, and intensity thresholding is then used to define the boundaries of individual filaments. Note the rapid rise in intensity at approximately 30 seconds. The increase is due to the Tpm1.1 which was bound to the actin filament. The intensity plateaus as the entire actin filament is coated with Tpm1.1. (B) The total intensity of the ROI as a function of time during the dissociation of Tpm1.1 from the same actin filament. The intensity profiles in panels A. and B. correspond to the kymograph shown in the panel C. The tropomyosin was loaded into the microfluidics channel at (C) 62.5nM, (D) 250nM, and (E) 500nM during the association phase. After full saturation of the actin filament the channel was washed with buffer only (dissociation phase). The vertical axes of each kymograph show the length of the filament where an individual pixel represents 92nm. The frames (pixels) shown in the horizontal axis were typically recorded at 1Hz for the 62.5nM association and 10Hz at the other concentrations. The magenta dashed line shows the boundary between the association and dissociation phases. The scale bars show 20 pixels (1.84 μm) in length. The yellow circle at approximately 64 seconds, at pixel 10 of the filament in panel C indicates the initial nucleation of binding.

30 filaments at varying Tpm1.1 concentrations were analysed (S1 Dataset kymographs and detected boundaries). Typical kymographs, containing both association and dissociation phases, are shown in Fig 3.

### Kymograph analysis

Several features are clearly visible in the kymographs (Fig 3). These include binding nucleations, where the Tpm1.1 first binds to an otherwise unbound region of the actin filament, such as occurs at approximately 64 seconds, pixel 10, of the association phase (Fig 3C, the yellow circle). Another nucleation event can be observed in the same filament at pixel 38, 88 seconds. The kymograph shows growth of the Tpm1.1 strand in both directions from these nucleation points, with further binding of Tpm1.1 next to nucleation points elongating the bound Tpm1.1 strand. The actin filaments in this study (see S1 Dataset) indicates that nucleation of Tpm1.1 occurs randomly on both sides of the filament. Additionally, the number of nucleation points increases with higher concentrations of Tpm1.1 as illustrated in Fig 3, panels C, D and E. We can also observe “meeting points” at which the elongation of the bound Tpm1.1 filament stops, such as at pixel 6, 84 seconds (Fig 3C), where two Tpm1.1 strands that elongate towards each other on the same groove of the action filament meet. This may result in a complete strand as illustrated schematically in Fig 1A. It may be, however, that two bound sections of Tpm1.1 are separated on the actin filament by less than the length of a single Tpm1.1 isomer. In this case a gap (a short stretch of undecorated actin filament with a length of 1–6 actin monomers) may remain between the two Tpm1.1 sections, Fig 1B. Actual gap points would likely be unobservable in the kymograph, due to the point-spread-function of the TIRF microscope and pixelation of the camera image.

The elongation of Tpm1.1 filaments towards one another can also be observed in Fig 3C at pixel 31, 124 seconds. In this instance, however, we believe that this does not indicate a meeting point. Rather, given the intensity pattern in later frames, this provides evidence of binding in two separate grooves of the actin filament, as discussed below.

In the dissociation phase, when Tpm1.1 is not present in the flow, the bound Tpm1.1 slowly dissociates from the actin filament. The converse of nucleation can be observed, for example, in Fig 3C at approximately pixel 5, 289 seconds, where the last bound Tpm1.1 dissociates from the actin filament.

### Intensity clusters

The kymograph intensities of the filaments obtained under the conditions described above show three distinct peaks: the completely unbound state (background level), a single bound level (one actin groove having bound Tpm1.1) and a double bound level (both actin grooves having bound Tpm1.1).

**Fig 4:**
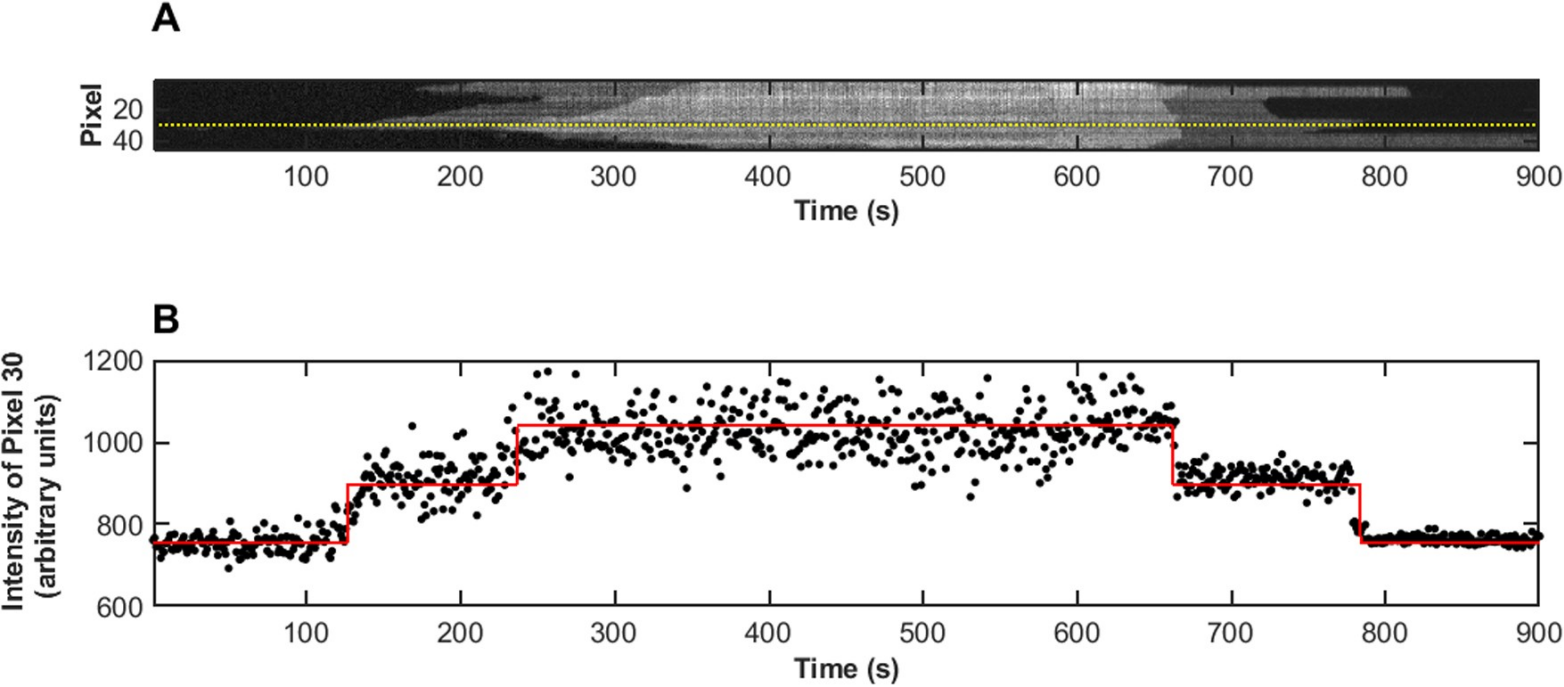
Two distinct Tpm1.1 intensity levels on an actin filament. (A) Kymograph, with pixel 30 indicated by the dotted line, and (B) intensity profile at pixel 30 of the filament shown in Fig 3D. The red line shows the fit to the intensity profile of pixel 30 of a function with identical magnitude intensity transitions. The transition magnitudes were found to be consistent across all the pixels in the kymograph. The fit optimised both the magnitude and the timing, with the results showing the transitions aligned with the single and double bound levels.

Naturally, the values of individual pixels in the kymograph do not represent single bound tropomyosins–there will be spread in the intensity and partial coverage in any given pixel because of the point spread function and pixelation. Nonetheless the distribution of intensities shown in Fig 3 indicates the feasibility of pinpointing binding on independent strands within the actin filament. The two intensity levels are consistent with the actin filament being decorated on one major groove of the actin filament in the case of the low intensity level, and on both major grooves in the case of the higher level. Our prediction is further confirmed by each transition in intensity–background to low level, and low level to high level–being the same magnitude (Fig 4), repeatable across all the filaments studied (S1 Dataset).

Here, a function comprised of two step transitions of identical magnitude was fit to the intensity profile for each pixel within the kymograph (considering the association and dissociation phases separately), as illustrated in Fig 4. The transition magnitudes were found to be consistent across all pixels, and the magnitude and timing of the transitions also matched well with the user-curated boundaries as discussed below.

### Filament boundary detection

**Fig 5:**
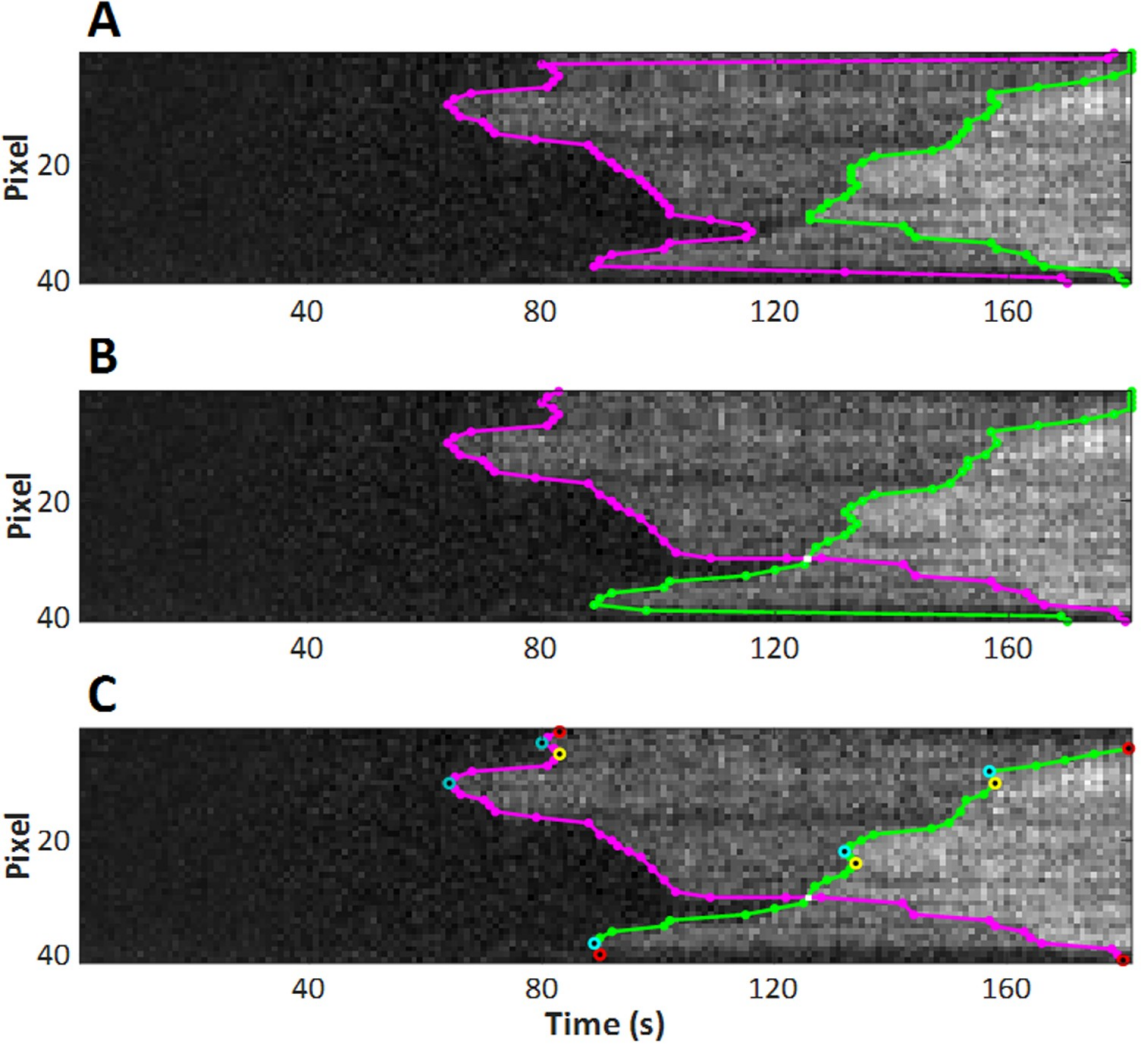
Crossing boundaries editing. (A) Boundaries as determined by intensity thresholding. The outer boundary occurs at the earlier time and lower intensity threshold (magenta) and the inner boundary is the later time, higher intensity threshold (green). (B) Boundaries after user editing. The boundaries have been cut and joined to show the likely cross-over–each boundary then indicating binding on different sides of the actin filament. (C) Nucleation points (light blue circles) and meeting points on the same groove (yellow circles) can be automatically detected at the extrema in each time-pixel boundary. Other points of interest can also be marked by the user to allow for other analyses.

The kymographs were analysed to detect the boundaries of the Tpm1.1 strands in each frame. This was done using a semi-automated process using custom-made Matlab (R2015b Mathworks 2015) based scripts. The kymograph was thresholded and the resulting binary image processed using a two-dimensional median filter, which eroded the boundary using a user defined structuring element. The binary image was then in-filled and re-dilated. We were able to alter the threshold and structuring element to best define the boundaries within the image–both the outer edge (i.e. between a naked region of the actin filament and a single decorated region of the actin filament) and any edges within the bound region of the kymograph (i.e. between single and double decorated regions of the actin filament). A single user (MJ) defined the boundaries for the experiments to ensure consistency. The thresholded edges were then minimally edited to account for any artefacts, such as fixed, non-specific fluorescence points not associated with the filaments (seen as fixed horizontal lines in the kymographs), and to account for points where Tpm1.1 strands growing towards each other on opposite sides of the actin filament cross over (Fig 5).

It can sometimes be ambiguous whether the boundaries cross over or whether a meeting point on one boundary is simply in alignment with a nucleation point on another boundary for the other side of the filament. An example of this situation can be seen in Fig 5, near pixel 31, 124 seconds (Fig 5A).

**Fig 6:**
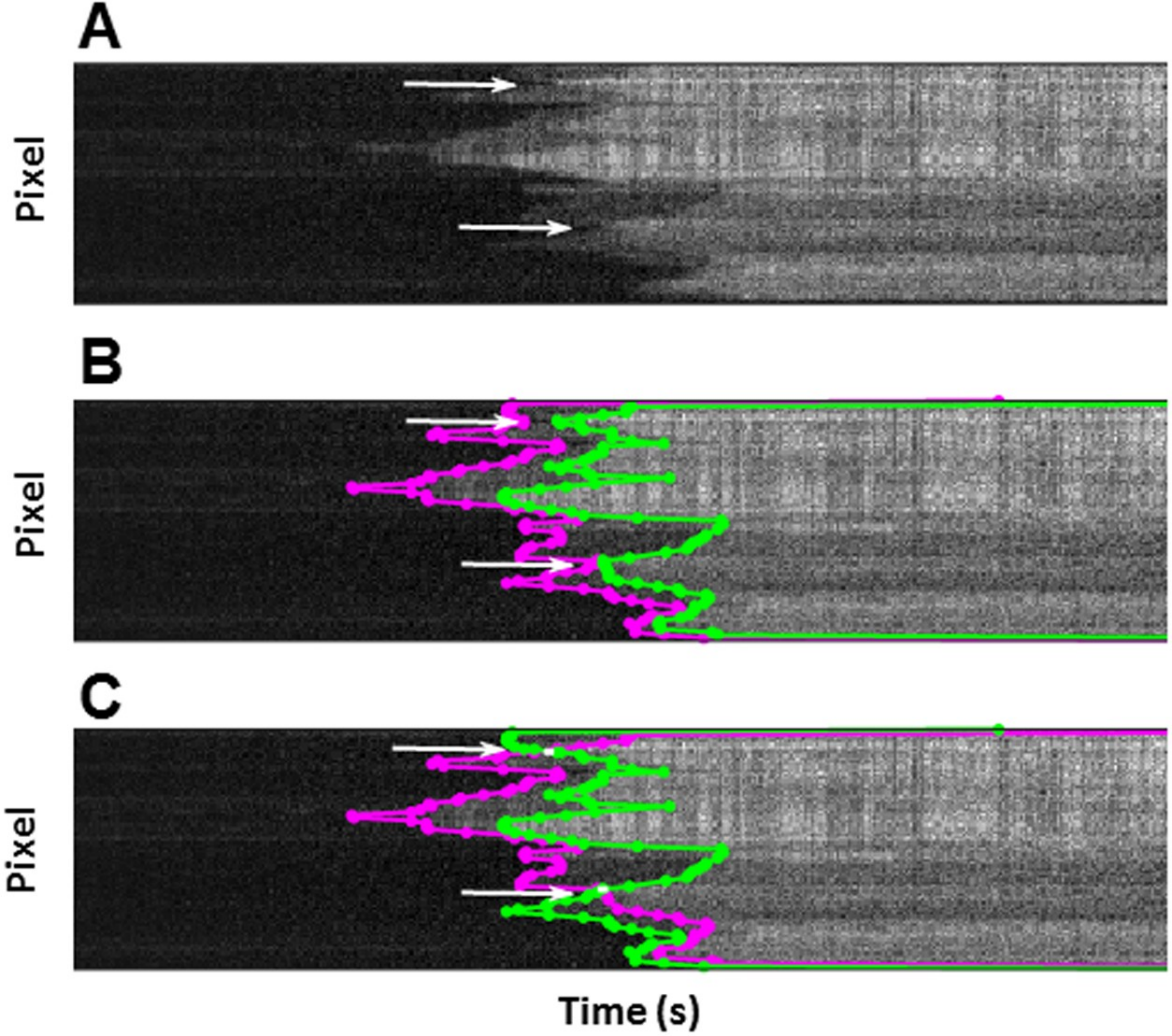
Identification and editing of crossing boundaries in complex kymographs. Processing of kymographs containing multiple nucleation points required the following general steps: (A) Identification of the crossing points; (B) detection of edges by intensity thresholding; and (C) the final editing generating, in this example, crossing points between boundaries.

An assessment of the gradient of the boundaries either side of the region may help indicate the likelihood of the two: should the crossed boundary have a similar gradient either side of the point then this reinforces the assumption that the point is where two tropomyosin filaments are elongating past each other on two separate actin grooves. The determination of whether the boundaries cross in case of the filament shown in Fig 5 is relatively straightforward. However, Tpm1.1 binding to longer actin filaments at higher concentrations (as shown in Fig 6) produces complex kymographs, where determination of crossing points could be more challenging. A quick and efficient estimation of the possible boundary crossing points is achieved by locating where a minimum in the time-pixel boundary between the background and singly bound regions meets a maximum of the time-pixel boundary between the singly and doubly bound regions, creating two opposite facing “V” shaped sections (indicated with arrows in Fig 6A). The meeting point of both “V” shaped sections is easier to identify after detection of boundaries (Fig 6B). Once potential crossing points are established, we can proceed with boundary editing (Fig 6C), as described above.

Once the boundaries were determined we compared the intensity transitions and timings identified by fitting the pixel intensity profiles (Fig 4) to the position and intensities of the regions defined by the boundaries. It was found that the timings determined from the fits corresponded well to the placement of the boundaries. This may seem unsurprising given the boundaries were obtained using intensity thresholding; however, the fitting constrained each of the transitions to have the same magnitude, the value and the timings of which were determined by a least-squares best fit of the pixel intensities to two identical step functions. The boundaries were used to define the background, mid-intensity and high level intensity regions, and the mean values for these regions determined. The transition magnitudes, from background to the mid-intensity level and from the mid-intensity to the high level, were determined as the difference in intensity between each region. Note that this evaluation did not constrain the transition magnitudes to be identical, as in the first method. The values determined via both methods were however in good agreement for all the kymographs analysed. The values of the transition magnitudes determined using the boundaries varied from kymograph to kymograph. Each pair of transition magnitudes (Background to mid-level and mid-level to high-level) was similar however. The association kymograph transition magnitudes were different by 12% on average and the dissociation kymograph transition magnitudes were a little more variable at 34%, averaged across all the kymographs. (The percentage difference was determined as the difference in the two transition magnitudes relative to their mean in each kymograph. The association and dissociation phases were analysed separately). This again reinforces the idea that the different intensity regions in the kymographs correspond to single and double bound regions of the actin filament.

Key points of interest in the kymographs during the association phase include the nucleation points, the points at which Tpm1.1 initially binds to an otherwise unbound region of actin, and meeting points, where the two strands either join (if they are in register with each other) or leave a gap of one to six actin subunits (a single Tpm1.1 dimer covers 7 actin subunits). Nucleation points can be determined from the boundaries detected in the kymographs as maxima in the time versus pixel boundary of the kymograph (Fig 5C).

Similarly meeting points can be automatically detected as minima of the time versus pixel boundary (Fig 5C). These points may be dissimilar than subjective placement of nucleation and meeting points in the absence of marked boundaries, as the human eye may use other information in determining the locations of these points. Determination of elongating filaments crossing on separate actin grooves can be subjectively different in the presence or absence of the thresholded boundaries on the kymographs. We believe, however, that using the quantitative boundaries provides robust measures of the filament processes.

In the dissociation phase, dissociation end points (DEP, corresponding to tropomyosins that are last to dissociate with a shrinking domain) can be similarly located as minima in the time versus pixel dissociation boundaries. As with the association phase, two distinct intensities were visible across all the kymographs investigated in the study, indicating Tpm1.1 bound in one or both grooves of the actin at the same point along the filament (S1 Dataset). Similar to the analyses for the association phase, points where the Tpm1.1 first dissociates from the filament, dissociation initiation points (DIP) can be located as maxima in the time versus pixel dissociation boundaries. These points could also be independently identified, without reference to the quantitative boundary identification.

**Fig 7:**
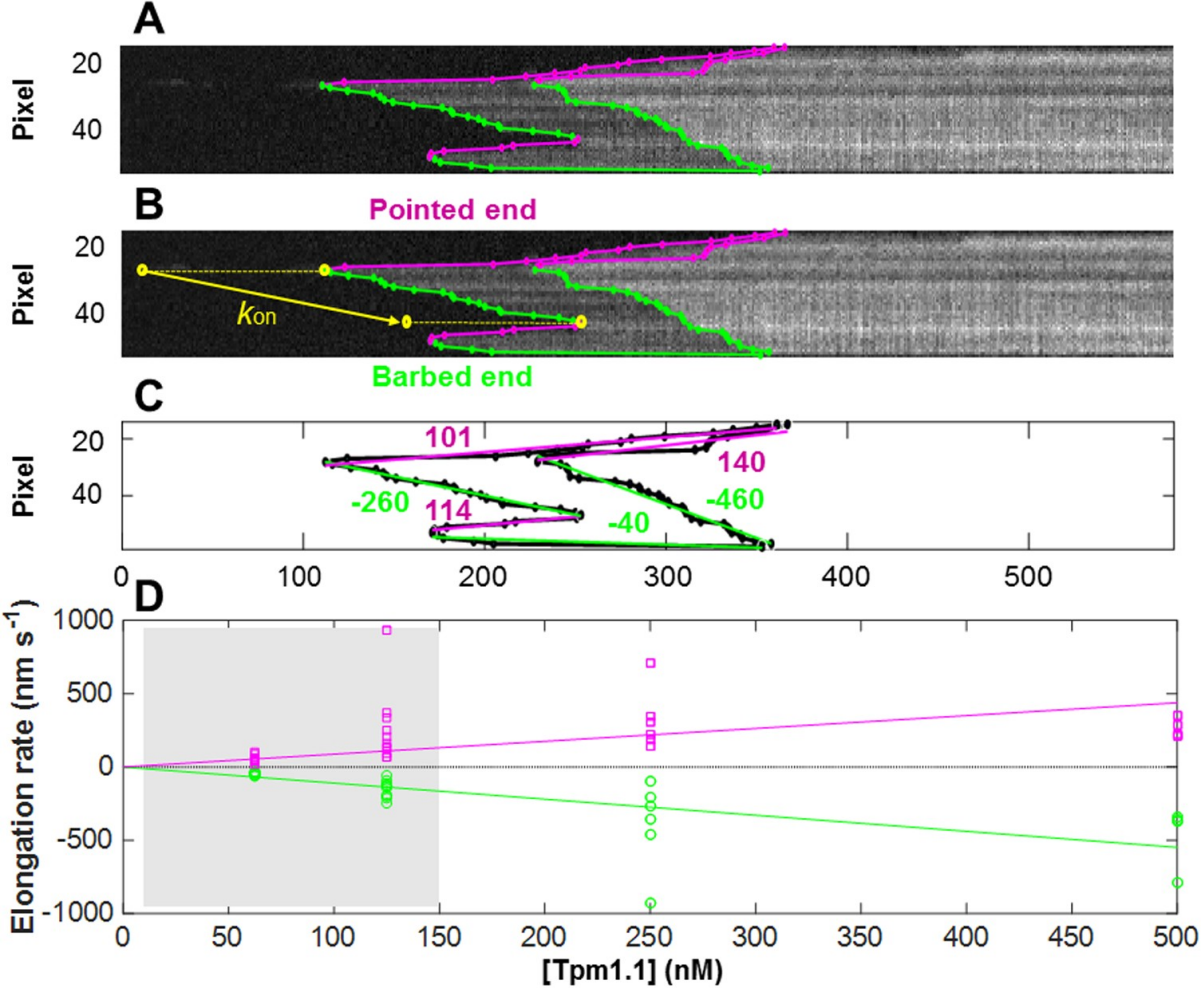
Estimation of elongation rates from edges of kymograph. (A) Kymograph of the AF647-Tpm1.1 nucleation and elongation on single actin filament. Two levels of intensities represent separate Tpm binding on two sides of the filament. (B) Edges of the kymograph were detected as described in the text. Green and magenta edges show individual Tpm1.1 elongation stretches towards barbed and pointed end of the filament, respectively. The yellow arrow demonstrates the average Tpm1.1 elongation rate extracted by linear fits to the kymograph boundary data. (C) Individual rates for each Tpm1.1 elongation segment on both grooves of actin filament. The weighted average elongation rate towards the barbed end was 136 nm s^-1^ and towards to pointed end 104 nm s^-1^ for the filament edges in Fig 3B. (D) Tpm1.1 elongation on actin towards opposite ends of the filament as a function of the feed concentration for all filaments (S1 Dataset). The lines show linear least squares fits constrained to go through the origin. These indicate that the elongation increases with the feed concentration at 1.1 nm s^-1^ nM^-1^ towards the pointed end and at 0.88 nm s^-1^ nM^-1^ towards the barbed end. The shaded area between 0 and 150 nM represents data points with slower protein delivery into the channel, which may have altered the actual concentration at which the association took place.

The elongation rate (in the case of association) and shrinkage rate (in the case of dissociation) of the Tpm1.1 strands can be determined from the gradient of the boundaries, either as an average between two points (Fig 7), or as a continuous measure along the length of the boundary. Measurement of the elongation rates on the single filament (Fig 7A) requires the initial determination of all kymograph boundaries. This process was followed by identification of the nucleation points and end points (Fig 7B). The average Tpm1.1 elongation rate was then estimated by the linear fit between the nucleation point and its neighbouring end points. This way, we extracted elongation rates for all nucleation points, elongating towards barbed and pointed ends of the actin filament, on both sides of the filament (Fig 7C). The average association rates for each filament were determined both towards the pointed (positive) and the barbed (negative) end of the filaments. The rates for the filaments in study (S1 Dataset) are summarised as a function of the Tpm1.1 feed concentration in Fig 7D. It can be seen that the association rates towards both ends were quite variable from filament to filament. On average, however, the rates in both directions were similar in magnitude and exhibited similar trends with concentration. Linear least-squares fits, constrained to pass through the origin indicate that the elongation increased with the feed concentration towards the pointed end at a rate of 1.1 nm s^-1^ nM^-1^ and towards the barbed end at 0.88 nm s^-1^ nM^-1^. It should be noted however, that at the lower concentrations (in the shaded region of Fig 7D), slow protein delivery into the channel means that the feed concentration may not be completely indicative of the concentration at which the association took place. This illustrates the need for a rapid switching system when investigating the low concentration dependence of the interactions.

The scripts written for the extraction of kymograph boundaries and the determination of points of interest and rates are available upon request from the authors.

**Fig 8:**
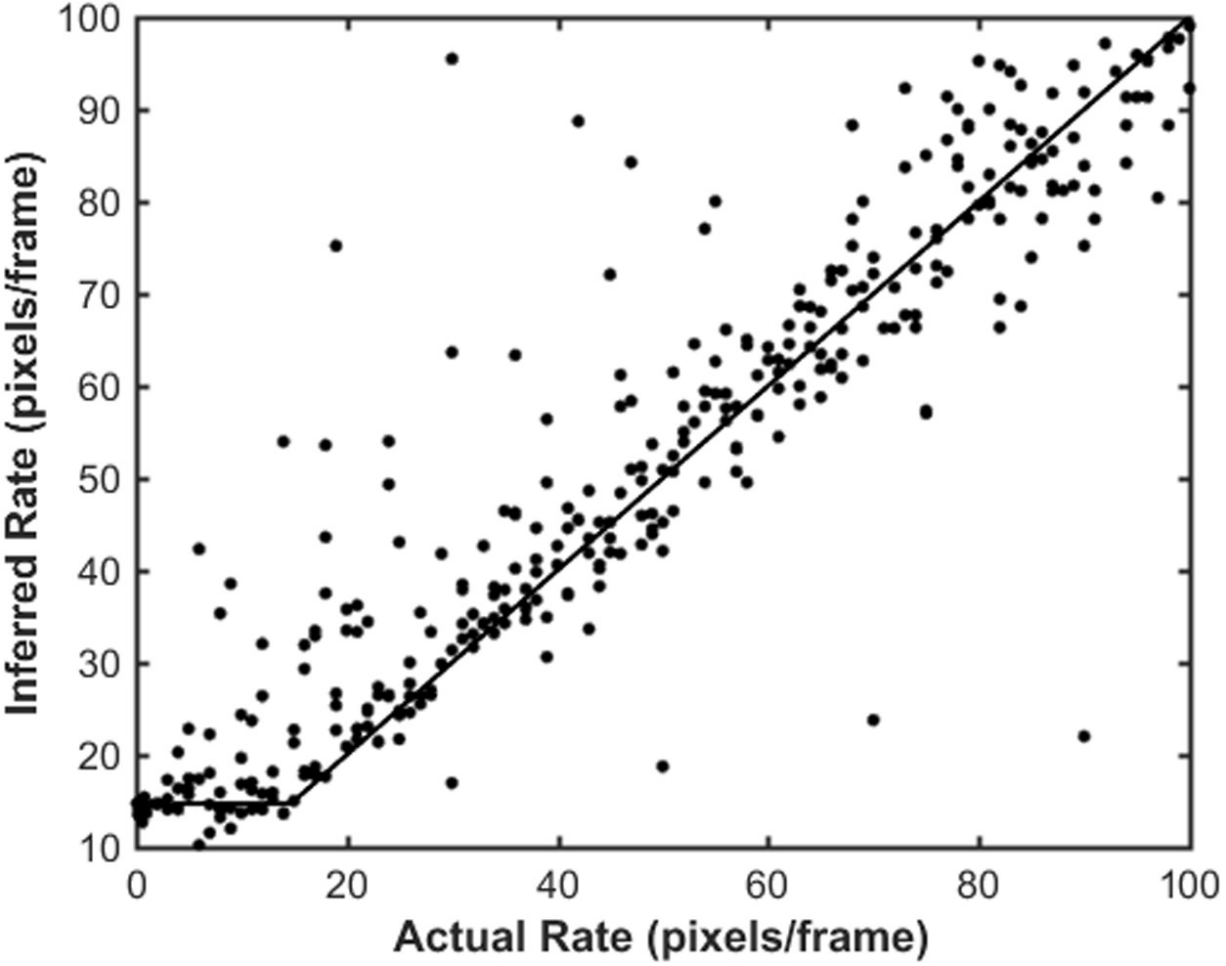
Inferred rate versus actual rate for simulated data. Movies of filaments elongating at different rates, 10^−4^ to 0.1 pixels/frame, were simulated. Kymographs were extracted, edges detected from the simulated data, and the inferred elongation rate determined in the same manner as the experimental kymographs. The data (points) was fitted to Eq (1) (line). For the example shown, the rate was inferred from segments of the boundary 10 temporal pixels in length, and the movie was simulated at a 0.1 Hz frame rate with 100 nm/pixel.

It is worth noting that all rate measurements derived from kymographs are limited by the resolution and pixelation of the microscopy images. Simulation experiments (Fig 8) were undertaken to create 400 image stacks of filaments growing at rates between 10^−4^ to 0.1 pixels/frame. The simulated data was generated by creating a single pixel wide filament of a specified length in an image stack. The image was convolved with a Gaussian kernel of sigma 2.5 pixels, typical of the single molecule microscope. Noise was then added to the image, as described previously [11].

The kymographs and boundaries of these simulated filaments were then extracted as outlined above and the rate of filament growth inferred from linear fits to the boundaries of the filament in the kymograph. As may be expected, the inferred rate was affected by the number of temporal frames over which the estimate is made. Rates were determined for segments from 1 to 50 temporal pixels in length. Over a large range of rates the actual and measured rates were in high correspondence, however for low rates the pixelation and resolution of the experiments caused the measured rate to be higher than the actual value. The cutoff rate, *R*_*cutoff*_, below which the measured rate was an overestimate, was determined by fitting the relationship between the actual rate, *A*, and the measured rate, *M*, as

$$M=R_{cutoff}+(A-R_{cutoff})H(A-R_{cutoff})$$ (1)

where $H(A-R_{cutoff})=\left\{ \begin{array}{c} 0,\;A-R_{cutoff}<0\\ 1,\;A-R_{cutoff}>0 \end{array}\right.$ is the Heaviside function.

The measured rates were determined using segments of the kymograph between 1 and 10 temporal pixels in length (reflecting the range of values observed for constant rates in the experimental kymographs). The cutoff value was found to be (1.48±0.05)×10^−2^ pixels/frame, independent of the segment length.

For point events, such as nucleations, rates can be inferred via multiple repeats across many filaments or experiments to record the time of nucleation relative to the arrival of Tpm1.1 in the flow (this requires rapid switching of the flow system to ensure relatively constant Tpm1.1 concentrations during the association phase). Alternatively, the nucleation rate can be inferred via analysis using Kaplan-Meier survival analysis [24]. For this approach, an estimate of the probability, *S*, that a nucleation will occur by time, *t*, is determined as

$$S(t)=\prod_{i<t}\left(1-\frac{d_{i}}{n_{i}}\right)$$

where *d*_*i*_ is the number of nucleation events that have happened by time *i*, and *n*_*i*_ is the number of available sites (pixels) at which nucleation events could occur at time *i*. The number of available sites for nucleation is the total length of the filament less those pixels either occupied by Tpm1.1 or those next to those pixels. The latter, neighbouring pixels, are not considered nucleation sites, but rather are points of possible elongation of an existing filament. *S*(*t*) is called the estimator.

**Fig 9:**
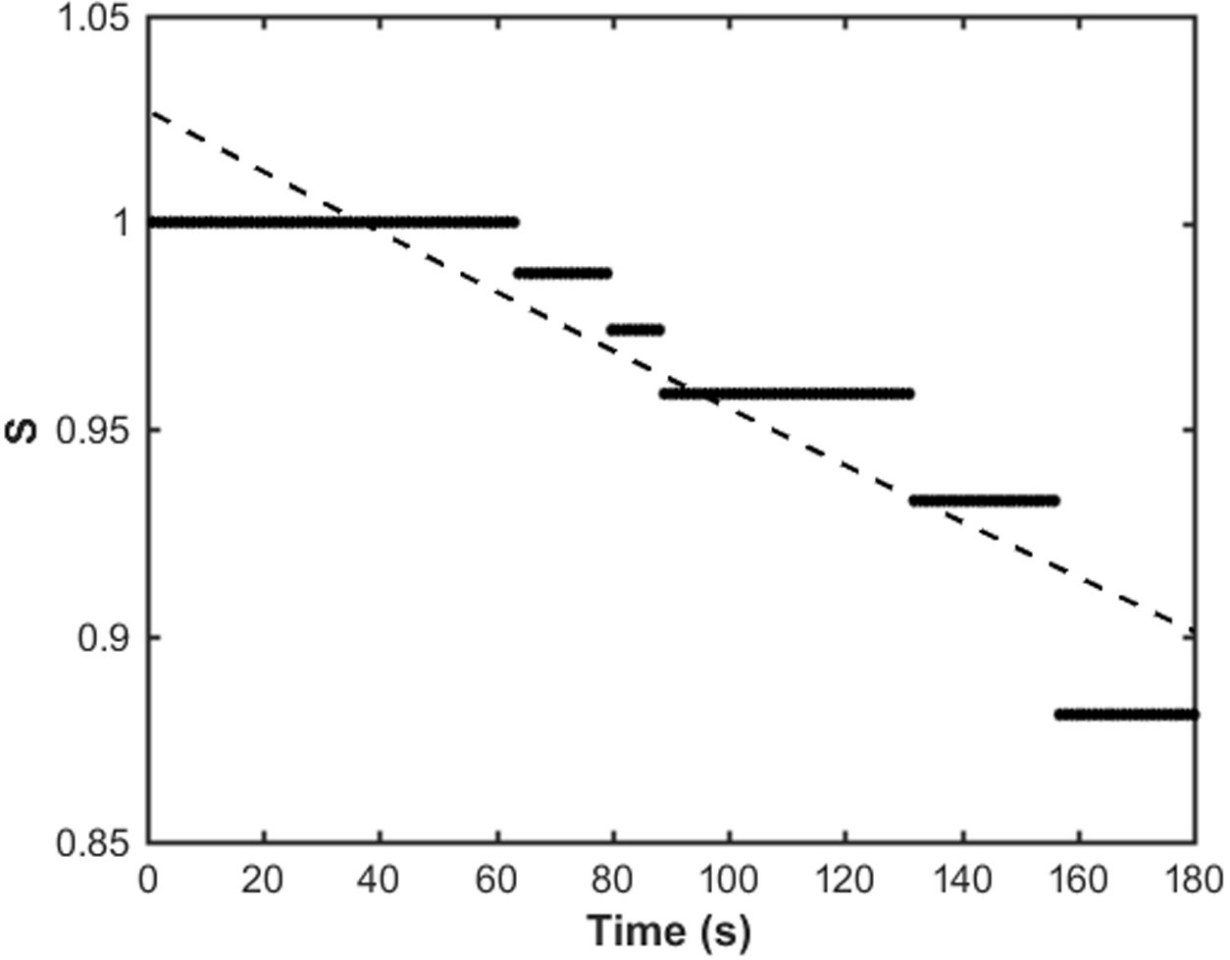
Example of the Kaplan-Meier estimate of nucleation time. The estimator data, *S*(*t*), for the filament are shown with black dots. The best fit exponential decay, $e^{-t/t_{nuc}}$, is shown by the dashed line, from which a nucleation time *t*_*nuc*_ ≈ 1370 seconds was determined. The Kaplan-Meier analysis here was done on the filament shown in Fig 3C.

The nucleation time, *t*_*nuc*_, is then determined from the best fit of the exponential decay, $e^{-t/t_{nuc}}$, to the estimator data, *S*(*t*) (Fig 9). If repeated measurements are made of filaments from the one movie, or from multiple movies under the same experimental conditions, the estimator data from each instance can be combined before fitting the exponential decay.

## Discussion

The development of a robust, quantitative and unbiased method for determining the boundaries between regions in kymographs of differing intensity allows for the development of high-throughput, statistically significant analyses of protein-filament binding events. Accurate boundaries can then be used, as demonstrated in Figs 5–7 and 9, to extract the timing and position of points of interest, the rates of elongation and dissociation. Furthermore, the boundaries can also be used to determine the regions of the filament that are bound at any given time, essential for, for instance, the inference of nucleation rates, Fig 9. The robust and quantitative determination of length and location of bound sections of the filaments also has ramifications for investigations into the locational specificity of the binding of specific isoforms and/or competition. Such studies require the accurate and repeatable identification of binding boundaries.

Naturally, due to experimental limitations, in particular the point-spread-function of the microscope, the edges may not be identical to the “true” edge of the bound state on the filament however the detected boundary will be systematically parallel to such an edge, and thus is still an accurate reflection of the changes taking place in the system.

We have identified the boundaries of binding and detachment of Tpm1.1 across 20 experiments under a range of concentrations (S1 Dataset). All the kymographs recorded indicated that distinct regions of single and double bound Tpm1.1 are observable. Using the guided intensity thresholding technique outlined above, the boundaries of the transition from unbound, to single bound to double bound were determined in a quantitative and robust way. The thresholding system did not constrain the user to specific values of intensity for placing the boundaries, however, upon analysis of the average intensity of the background, the region between the first and second boundaries (the intermediate intensity level, see Figs 4 and 5) and the brightest intensity region further confirmed the hypothesis that we were observing first single bound Tpm1.1 and then double bound Tpm1.1 on filaments (both grooves of the actin). All of the binding and unbinding experiments showed the same quantal step structure as the example shown in Fig 4. Typical features observed across the entire range of concentrations of Tpm1.1 experiments include the distinct, three level intensity regions from the differentially bound states, and also clear peaks and troughs in bound filament position versus time (Fig 5C), indicative of nucleation events and meeting point formation. The use of the extrema of the time-pixel boundary to locate these events provides a robust and independent means of identification. Naturally some of these auto-detected points may be erroneous, and we do not preclude the possibility that some extrema may not be actual filament binding events–especially in the case of rapid binding and/or bad signal to noise ratio datasets. An important caveat, especially in the automated analysis of large data sets to remember is that the resolution, both in space and time, of the data will also constrain the resolution of the information that can be extracted from the boundaries–as illustrated in Fig 8. The minimum association rate will however have a lower limit below which the true rate may be unobservable.

Binding on both sides of the actin filament is clear across a range of Tpm1.1 concentrations. It is also clear from our results that the binding dynamics are correlated with concentration, as expected in biological systems. Nucleation of Tpm1.1 appears to randomly occur on both sides of actin filament, similar to *S*. *pombe* tropomyosin Cdc8 [1], but different to observations for Drosophila tropomyosin Tm1A which showed preferential binding to the pointed end of the filament, and participation in the cofillin regulated formation of the lamellum [17]. Differences in the binding modes between tropomyosin isoforms could be caused by (i) the size and (ii) the sequence of the molecule, (iii) overlapping regions between two dimers, (iv) the nucleotide state on actin filament, (v) additional protein/s bound to actin filament, and (vi) potentially forces generated on actin filament in the cellular microenvironment. It seems that different modes of binding between individual tropomyosin isoforms are finely tuned by combination of these factors and the outcome heavily depends on cellular function. Therefore stochastic association of Tpm1.1 to highly organized and mostly rigid actin filament bundles in the muscle tissue in this study is not unexpected.

Despite the possibility of non-constant concentration during change-over during these experiments, a clear increase in the rate of attachment was observed with increasing concentrations of Tpm1.1, with the double bound regions occurring more rapidly following the singly bound levels with concentration. This indicates that the experimental setup needs to be tuned, in order to rapidly control the change-over from one fluid-flow concentration to another, if the concentration dependence of binding and dissociation features is to be determined. However none of these characteristics can be extracted with statistical significance without a robust and repeatable method of boundary extraction, such as that presented here.

Our study demonstrates an in-depth characterization of Tpm1.1 interaction with preassembled actin filaments. We have chosen this simplified system to elucidate important questions regarding (i) the mechanism of gap healing (which leads to the fully saturated, and therefore functional filaments), (ii) potential cooperative behaviour between tropomyosin molecules binding to the opposite sides of the filament, (iii) determination of kinetic parameters of Tpm1.1 on opposite sides of the filament. The rate of Tpm1.1 exchange on the filament by non-invasive methods could also be extracted using similar analyses to those presented, and will be addressed in detail in a future study. Recently it has been observed that cytoskeletal tropomyosin Tpm3.1 assembles to actin in parallel with actin polymerization [25], suggesting a lack of tropomyosin-free actin filaments in secretory granules of *in vitro* mouse model during exocytosis. Another study reported that actin-tropomyosin co-polymers represent major fraction of the actin cytoskeleton in a variety of human cell lines [26]. Note that the method and analyses as well as our experimental set up is able to investigate actin-tropomyosin co-polymerization, which will be used for future studies describing cytoskeletal isoforms.

The methods presented above demonstrate the means by which quantitative, semi-automated analyses may be made of large studies of protein-protein interactions, opening the way to dissect the dynamics underlying the interactions. The framework developed here, to quantify the association and dissociation rates, allows us to plan future studies to determine the precise concentration dependence of these processes, as well as the nucleation rates and dissociation characteristics.

## Conclusions

In this study we have demonstrated an analysis methodology that allows the quantification and interpretation of tropomyosin Tpm1.1 interactions with single actin filaments as measured by single molecule TIRF microscopy. We have clear evidence of multiple Tpm1.1 domains binding on actin filaments under a range of conditions. Due to our low level of Tpm1.1 labelling approach, we have been able to resolve two step intensity changes in the kymographs indicating the Tpm1.1 binding on opposite grooves of the actin filament. Individual boundaries from kymographs, for each side of the filament, were determined by intensity thresholding and used for accurate interpretation of kinetic processes of the system.

Based on kymograph boundaries, we have been able to extract nucleation points and rates, elongation rates of Tpm, identify meeting points after the saturation of filament, and when dissociation occurs, initiation point(s), the final dissociation point(s), as well as dissociation rates. All of these measurements were extracted from both sides of the filament, and therefore we have a system whereby we can compare possible differences of the behaviour on the two sides of the filament, and across concentrations. It remains the focus of a future study to quantify the specific concentration dependence of the system, for which we now have the analysis tools.

## Supporting information

S1 Dataset Kymographs for different concentrations of Tpm1.1. Boundaries are indicated on the lower kymograph by the lines, nucleation point by magenta circles, defects by yellow circles. In the cases where both association and dissociation phases are present, the change over from the association to dissociation kymograph is indicated by a dotted magenta line. The recording frame rate is indicated on the figure. All kymographs were recorded at 92nm/pixel.
(PDF)
