# Supplementary material for: Interactions of tropomyosin Tpm1.1 on a single actin filament: A method for extraction and processing of high resolution TIRF microscopy data
Source: PLoS One. 2018 Dec 10;13(12):e0208586. doi: 10.1371/journal.pone.0208586 (PMC6287813; doi:10.1371/journal.pone.0208586)
Supplement: S1 Dataset — Boundaries are indicated on the lower kymograph by the lines, nucleation point by magenta circles, defects by yellow circles. In the cases where both association and dissociation phases are present, the change over from the association to dissociation kymograph is indicated by a dotted magenta line. The recording frame rate is indicated on the figure. All kymographs were recorded at 92nm/pixel. (PDF) [file pone.0208586.s001.pdf]

1. 62.5nM, ROI 5a, 1Hz association

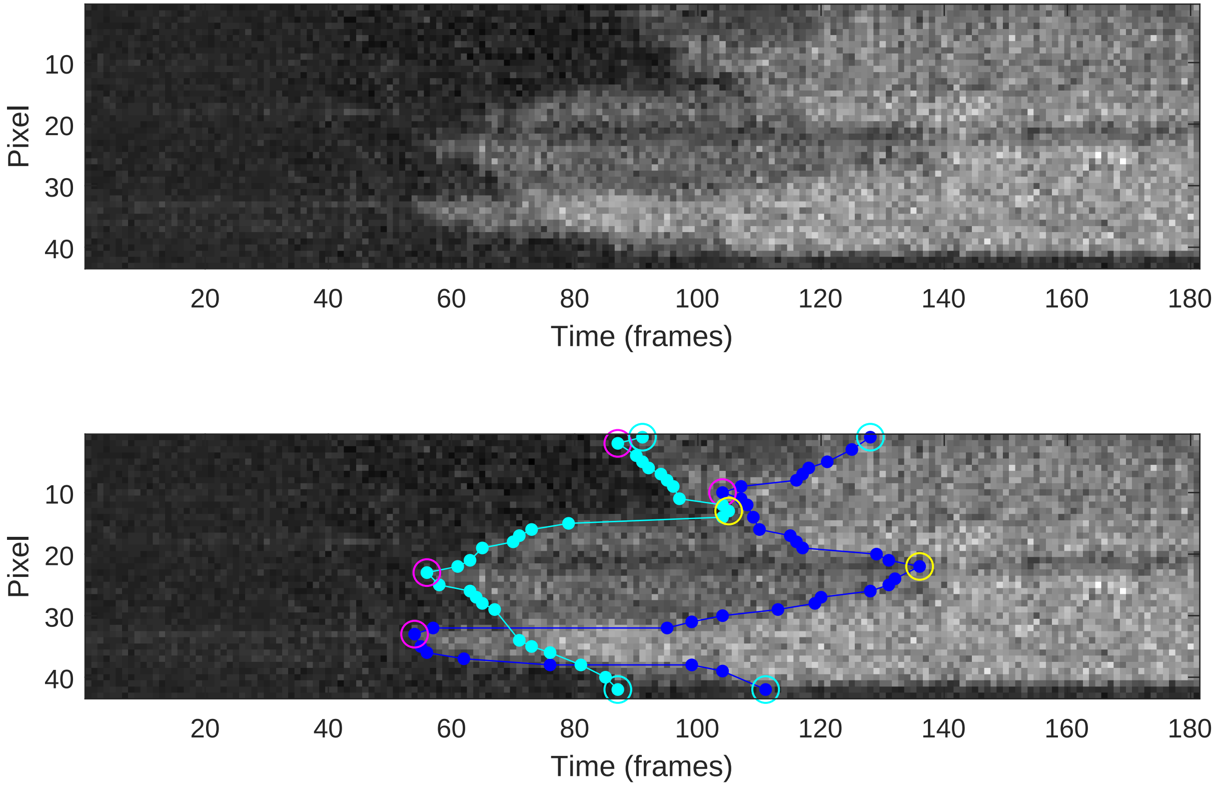

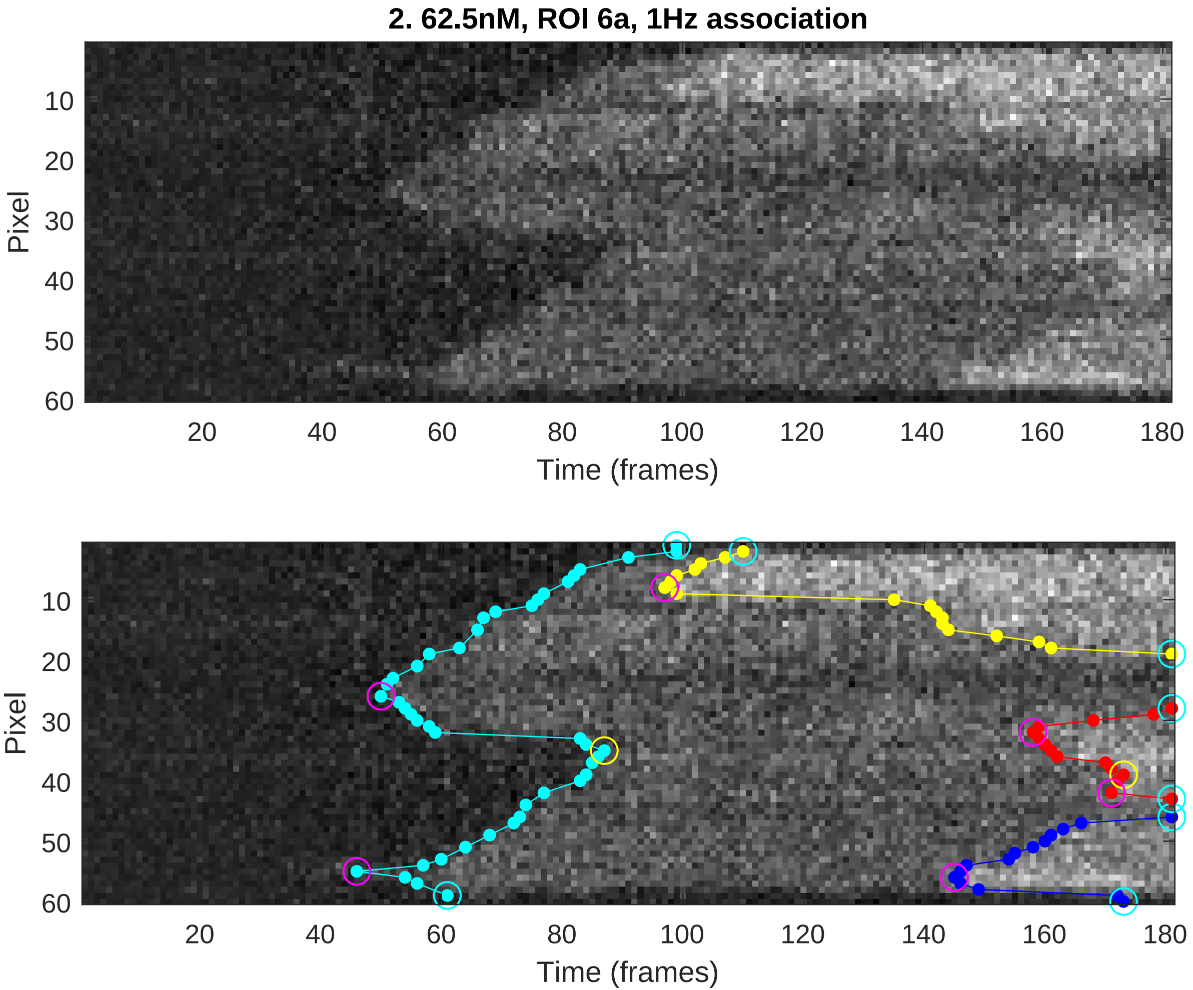
2. 62.5nM, ROI 6a, 1Hz association
Pixel
Time (frames)
10
20
30
40
50
60
20
40
60
80
100
120
140
160
180

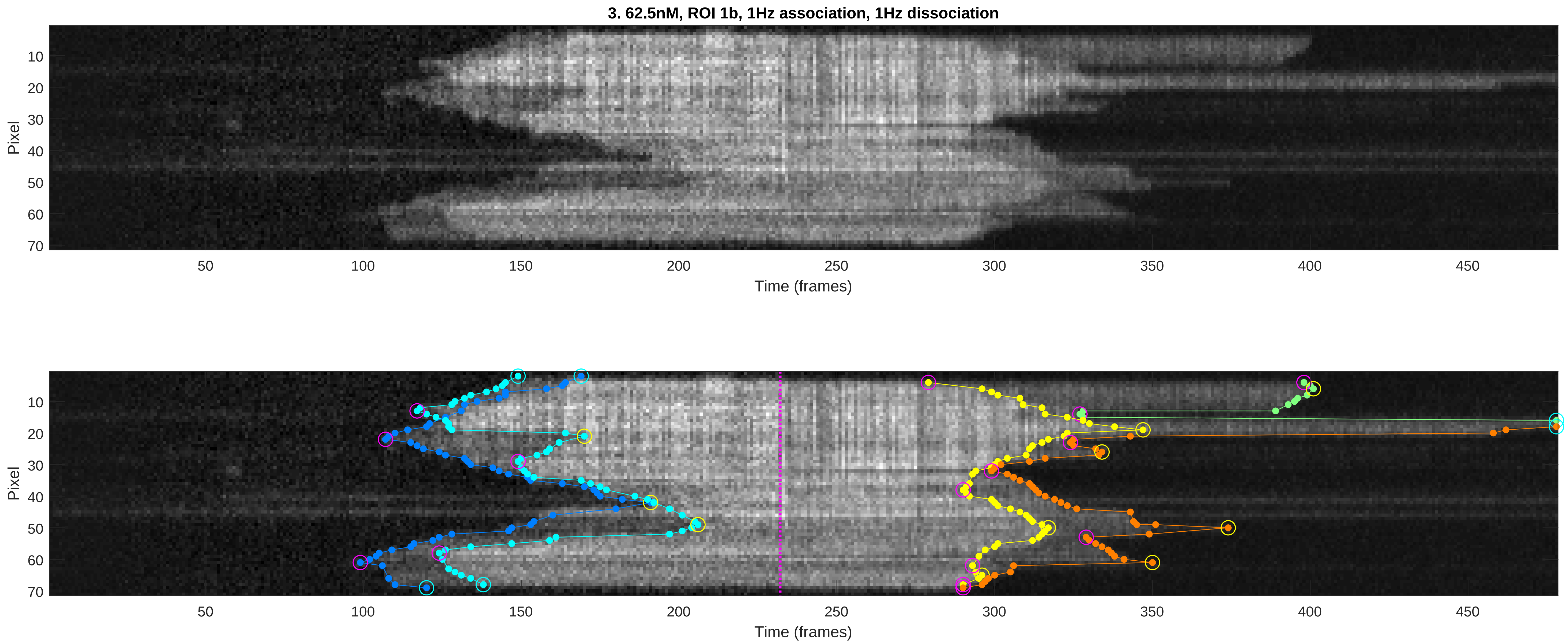
3. 62.5nM, ROI 1b, 1Hz association, 1Hz dissociation
Pixel
Time (frames)
10
20
30
40
50
60
70
50
100
150
200
250
300
350
400
450

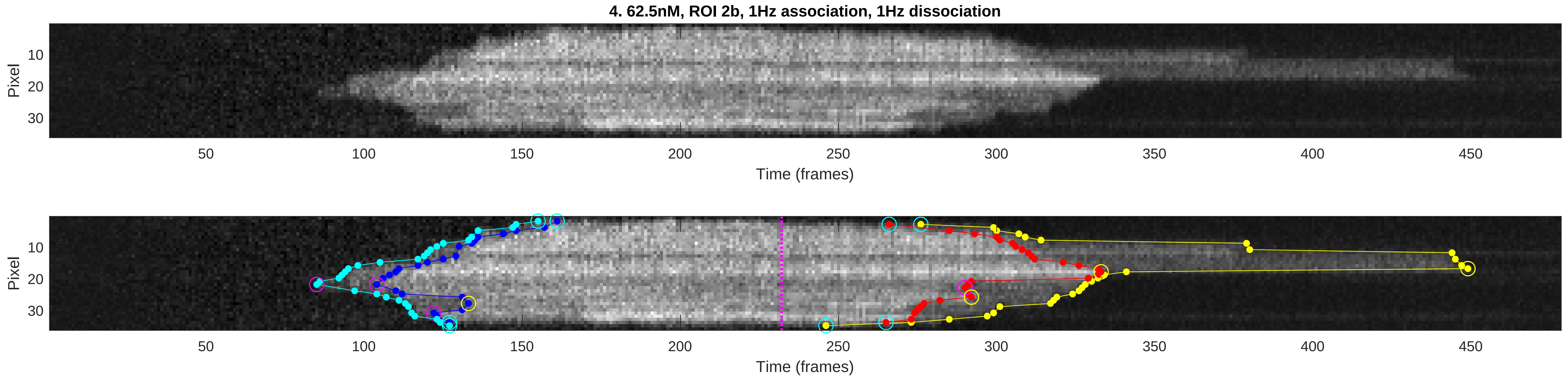
4. 62.5nM, ROI 2b, 1Hz association, 1Hz dissociation
Pixel
10
20
30
50
100
150
200
250
300
350
400
450
Time (frames)
Pixel
10
20
30
50
100
150
200
250
300
350
400
450
Time (frames)

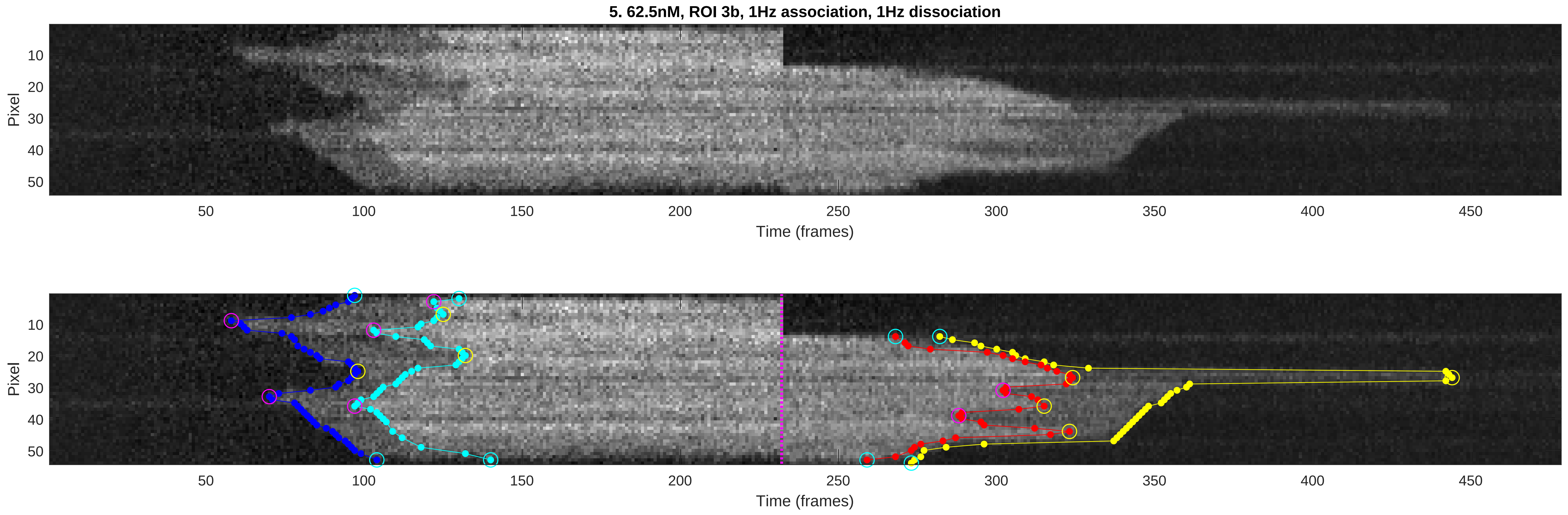
5. 62.5nM, ROI 3b, 1Hz association, 1Hz dissociation
Pixel
10
20
30
40
50
50
100
150
200
250
300
350
400
450
Time (frames)
Pixel
10
20
30
40
50
50
100
150
200
250
300
350
400
450
Time (frames)

6. 62.5nM, ROI 1c, 1Hz association, 1Hz dissociation

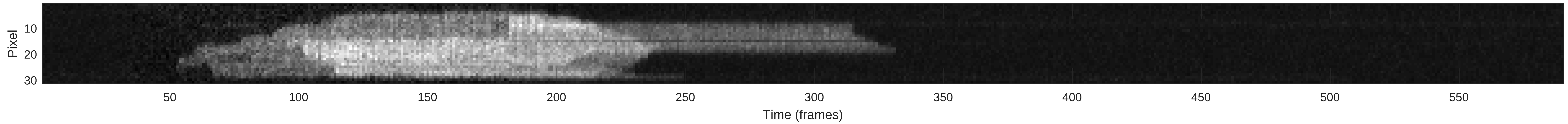

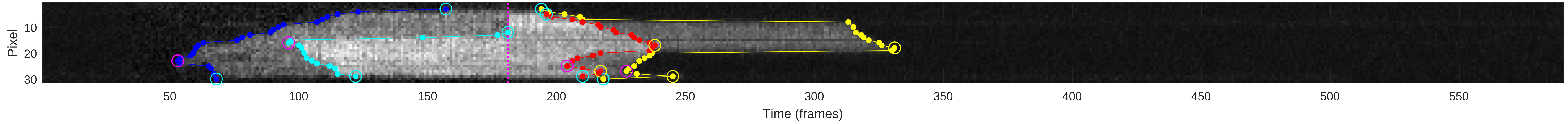

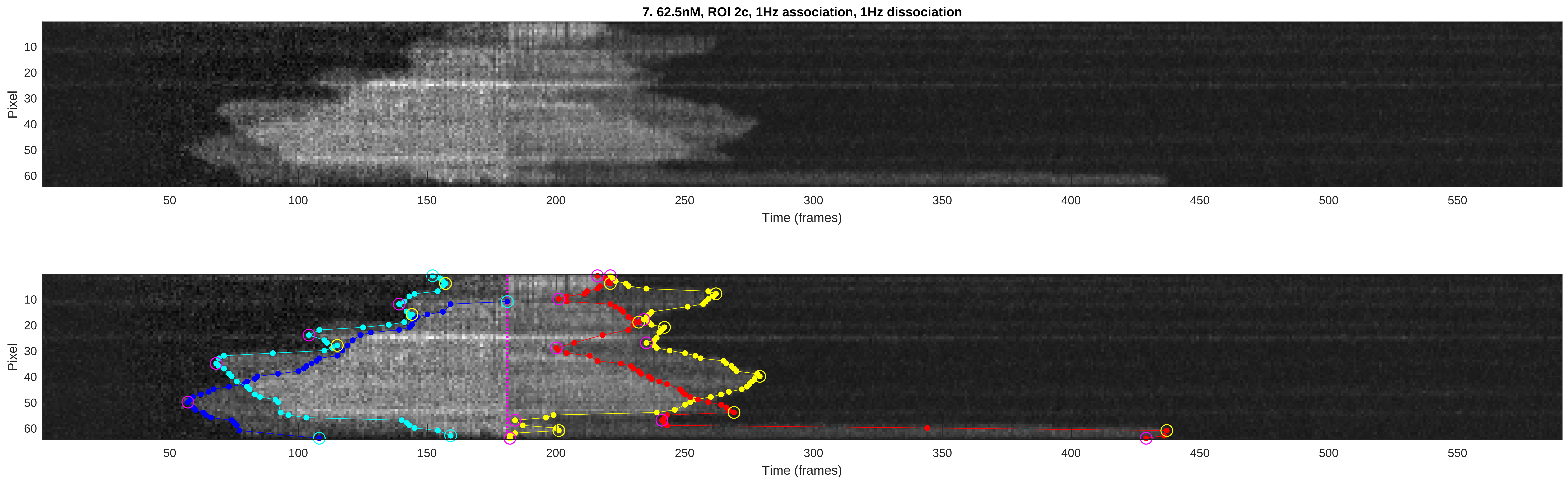

7. 62.5nM, ROI 2c, 1Hz association, 1Hz dissociation
Pixel
Time (frames)
Pixel
Time (frames)

8. 62.5nM, ROI 3c, 1Hz association, 1Hz dissociation

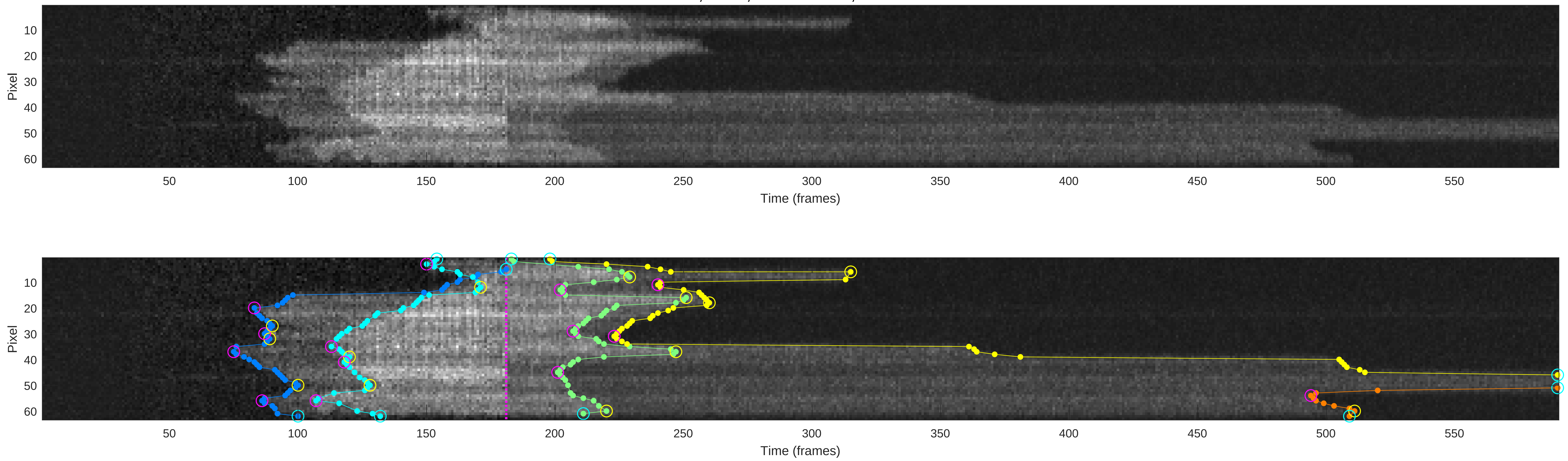

9. 62.5nM, ROI 4c, 1Hz association, 1Hz dissociation

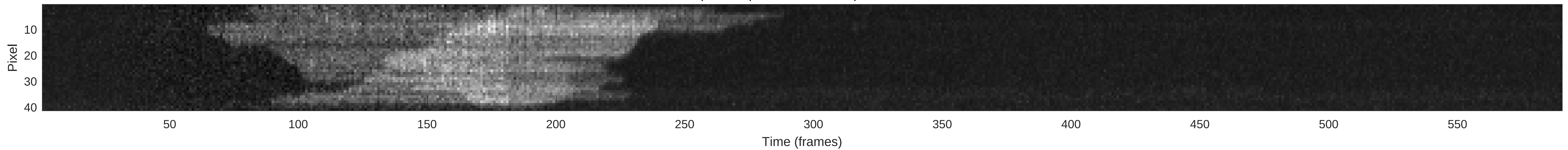

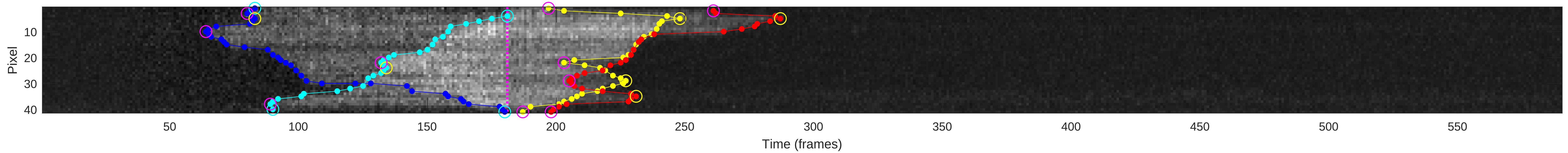

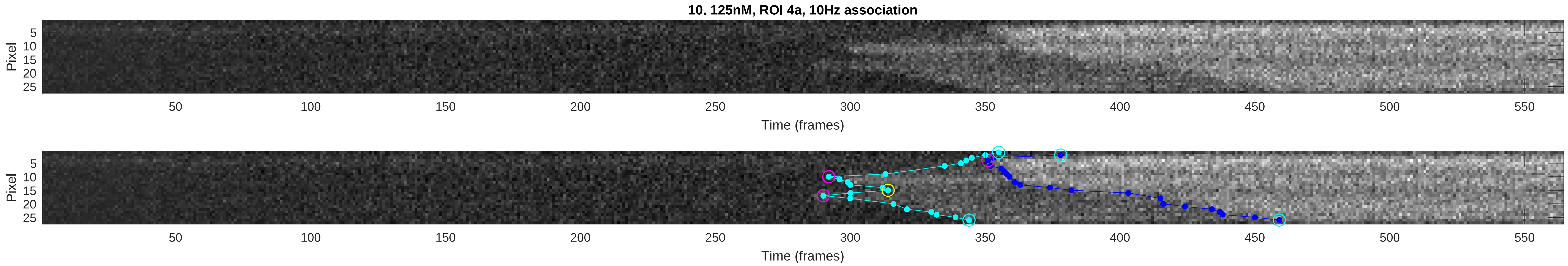

10. 125nM, ROI 4a, 10Hz association
Pixel
5
10
15
20
25
50
100
150
200
250
300
350
400
450
500
550
Time (frames)
Pixel
5
10
15
20
25
50
100
150
200
250
300
350
400
450
500
550
Time (frames)

11. 125nM, ROI 2b, 10Hz association

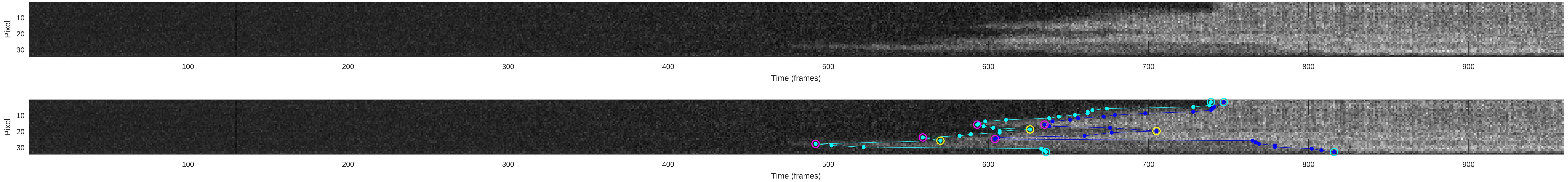

12. 125nM, ROI 3b, 10Hz association

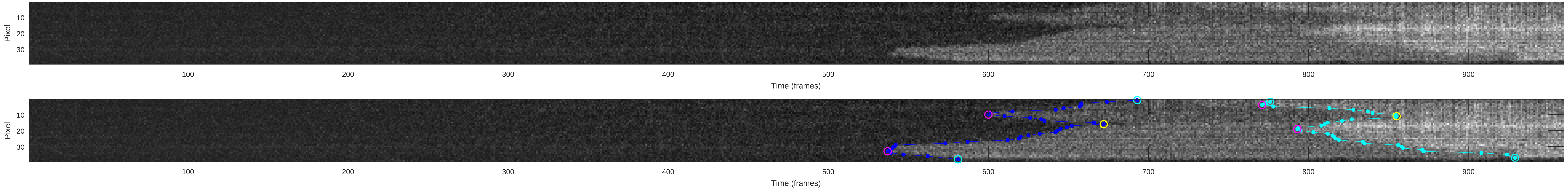

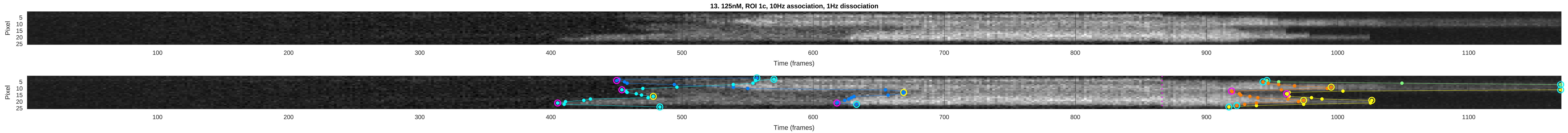
13. 125nM, ROI 1c, 10Hz association, 1Hz dissociation
Pixel
Time (frames)
Pixel
Time (frames)

14. 125nM, ROI 2c, 10Hz association, 1Hz dissociation

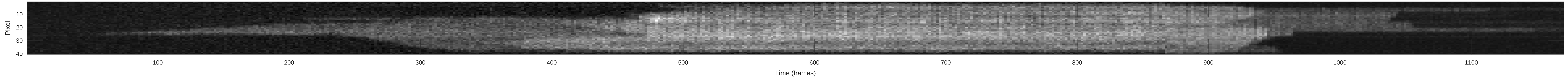

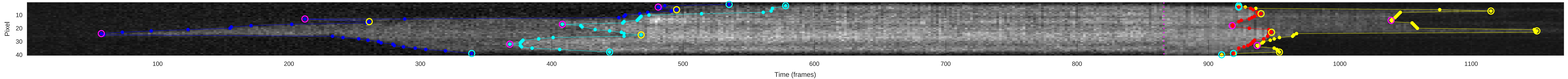

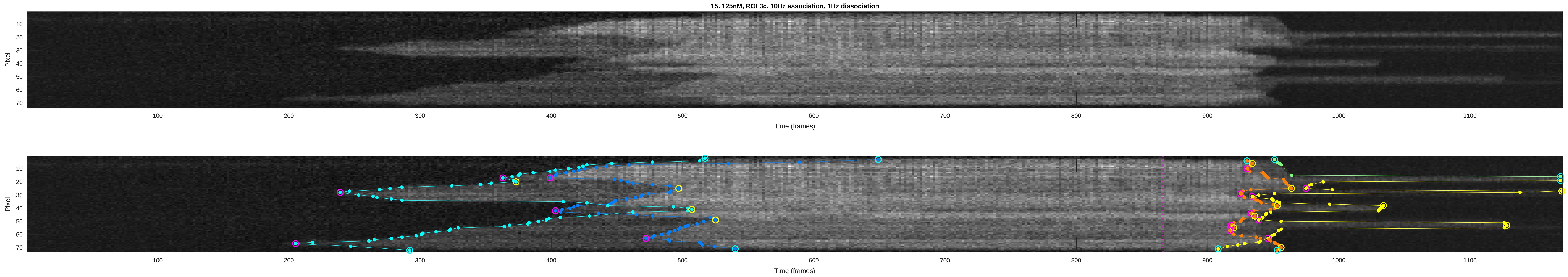
15. 125nM, ROI 3c, 10Hz association, 1Hz dissociation
Pixel
10
20
30
40
50
60
70
100
200
300
400
500
600
700
800
900
1000
1100
Time (frames)
Pixel
10
20
30
40
50
60
70
100
200
300
400
500
600
700
800
900
1000
1100
Time (frames)

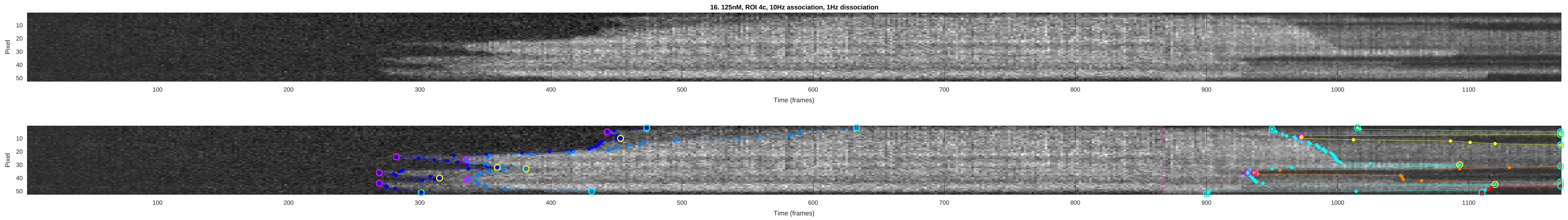

16. 125nM, ROI 4c, 10Hz association, 1Hz dissociation
Pixel
10
20
30
40
50
100
200
300
400
500
600
700
800
900
1000
1100
Time (frames)
Pixel
10
20
30
40
50
100
200
300
400
500
600
700
800
900
1000
1100
Time (frames)

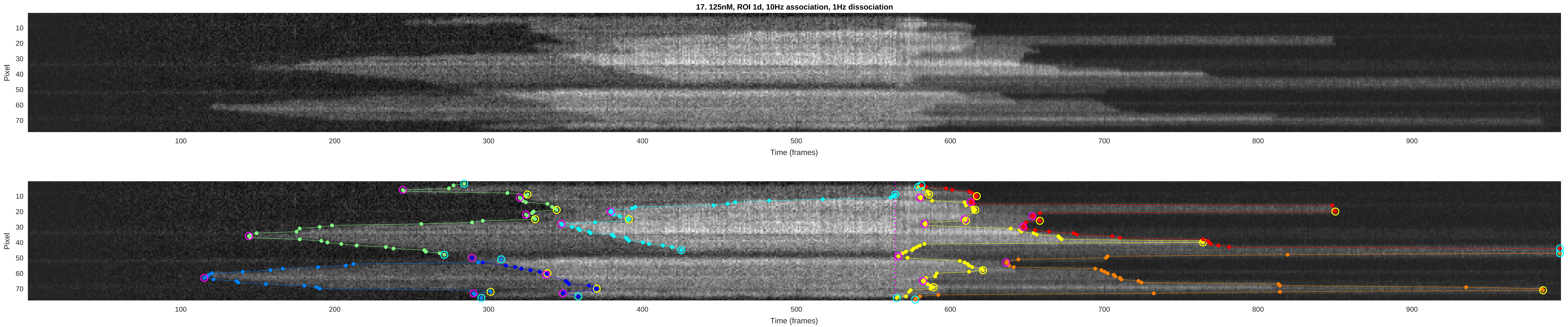
17. 125nM, ROI 1d, 10Hz association, 1Hz dissociation
Pixel
10
20
30
40
50
60
70
100
200
300
400
500
600
700
800
900
Time (frames)
Pixel
10
20
30
40
50
60
70
100
200
300
400
500
600
700
800
900
Time (frames)

18. 125nM, ROI 2d, 10Hz association, 1Hz dissociation

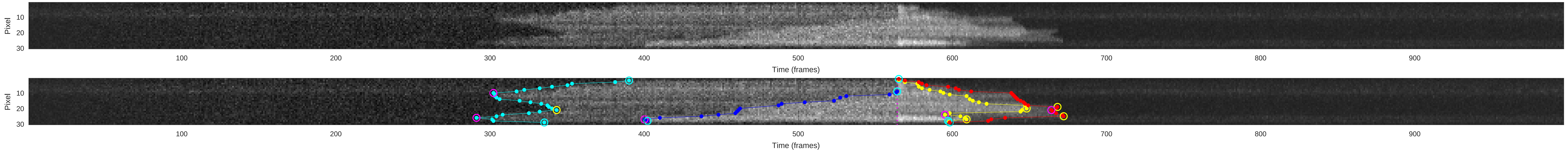

19. 125nM, ROI 3d, 10Hz association, 1Hz dissociation

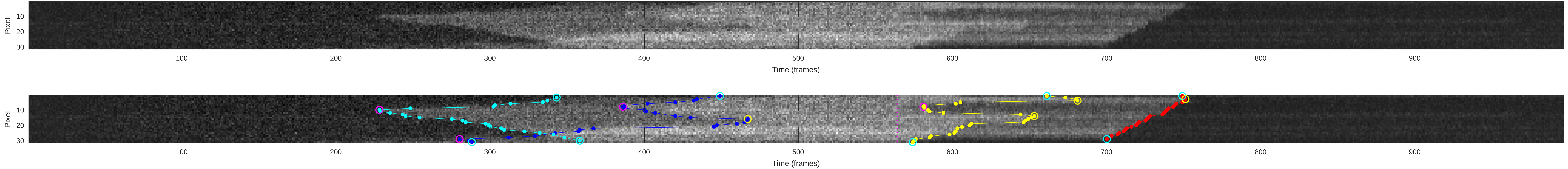

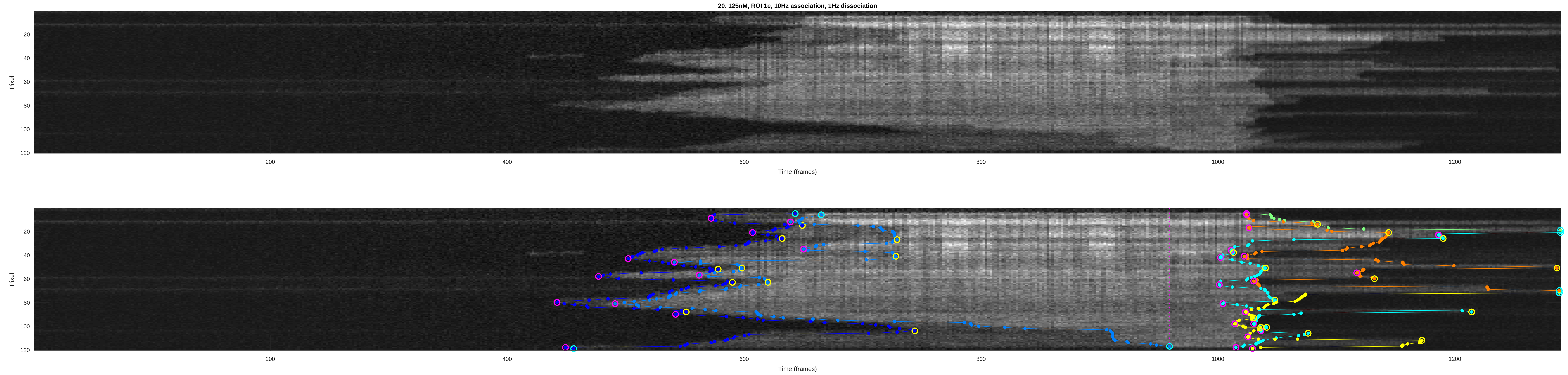
20. 125nM, ROI 1e, 10Hz association, 1Hz dissociation
Pixel
20
40
60
80
100
120
200
400
600
800
1000
1200
Time (frames)
Pixel
20
40
60
80
100
120
200
400
600
800
1000
1200
Time (frames)

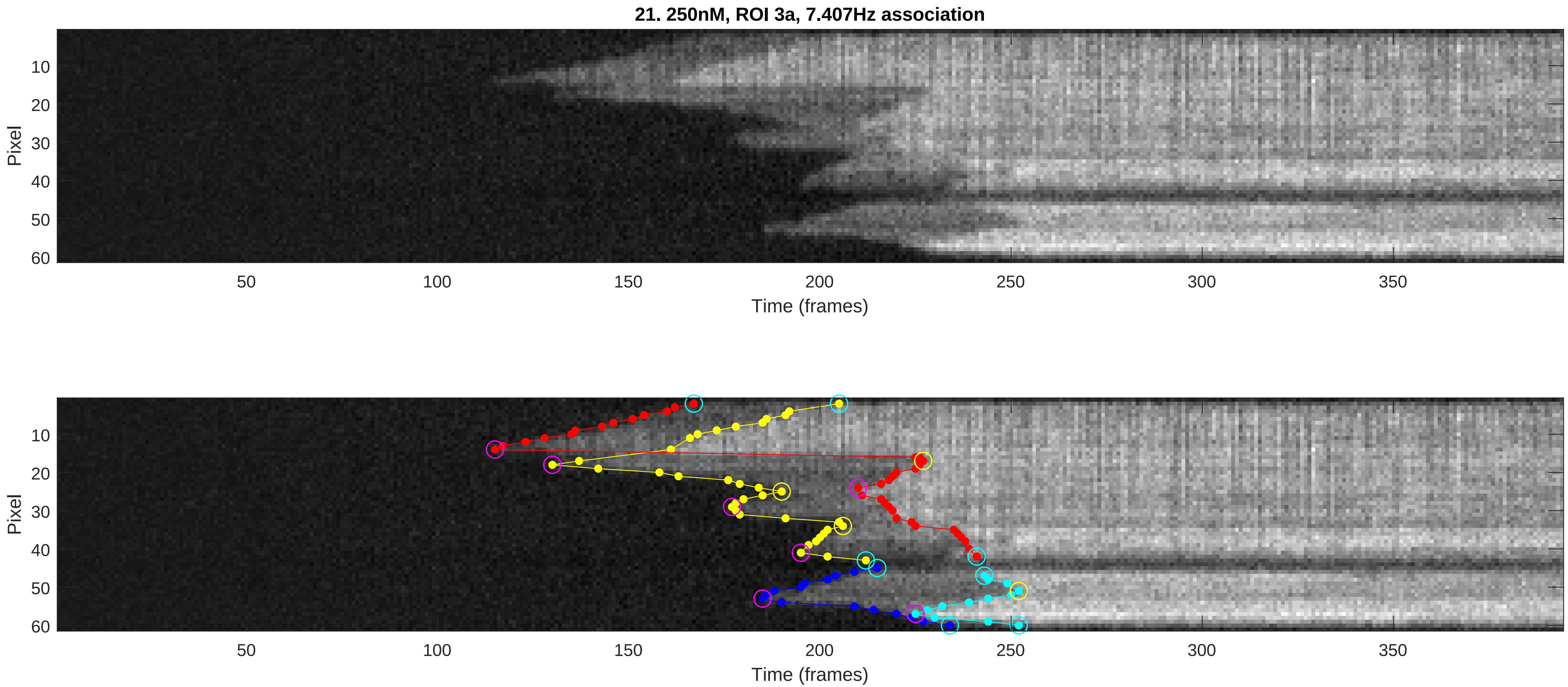
21. 250nM, ROI 3a, 7.407Hz association
Pixel
10
20
30
40
50
60
50
100
150
200
250
300
350
Time (frames)
Pixel
10
20
30
40
50
60
50
100
150
200
250
300
350
Time (frames)

22. 250nM, ROI 1b, 11.765Hz association

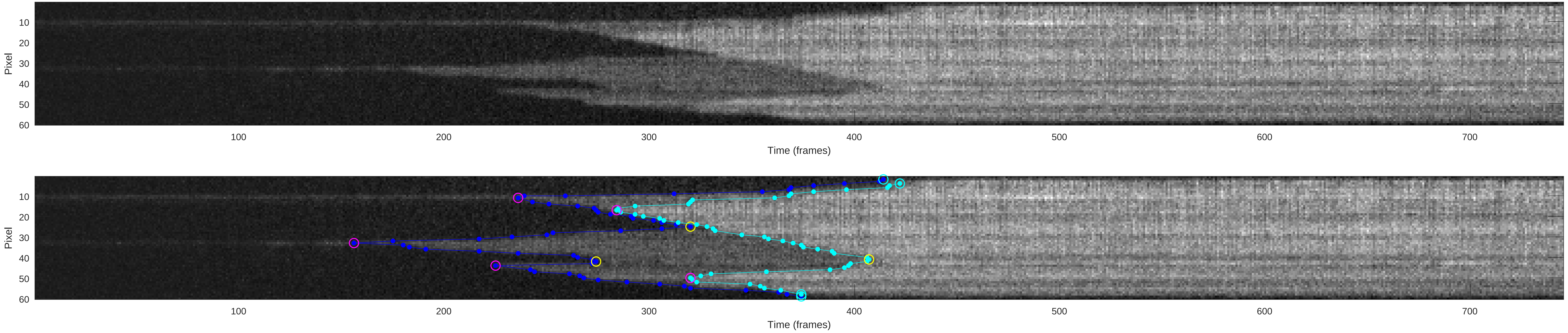

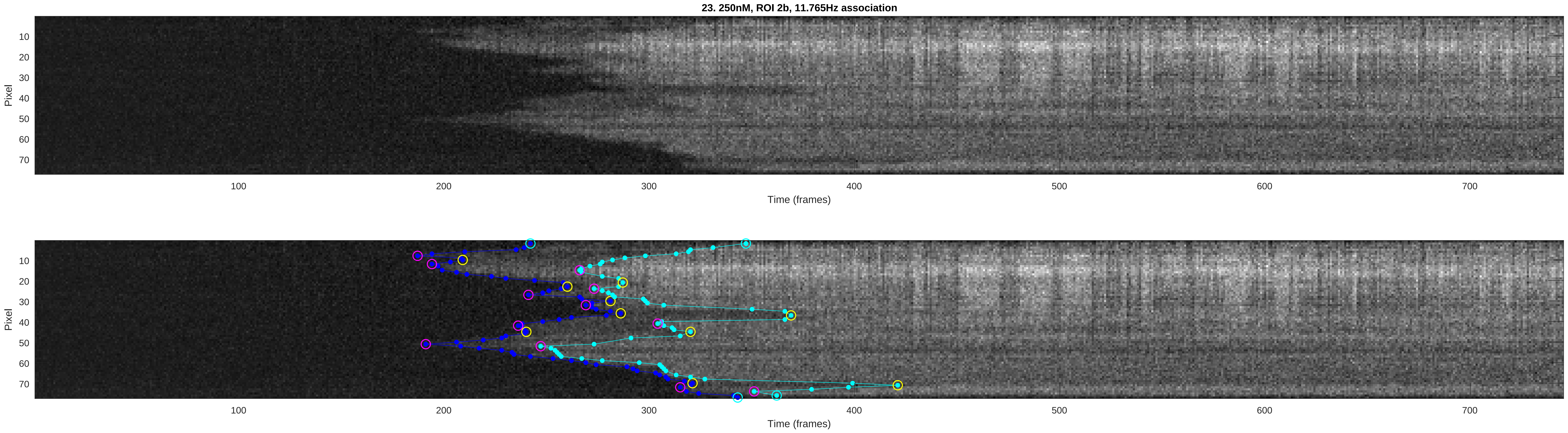
23. 250nM, ROI 2b, 11.765Hz association
Pixel
10
20
30
40
50
60
70
100
200
300
400
500
600
700
Time (frames)
Pixel
10
20
30
40
50
60
70
100
200
300
400
500
600
700
Time (frames)

24. 250nM, ROI 2c, 10Hz association, 1Hz dissociation

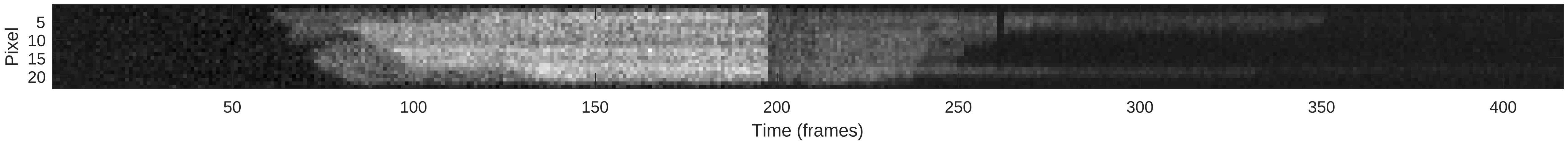

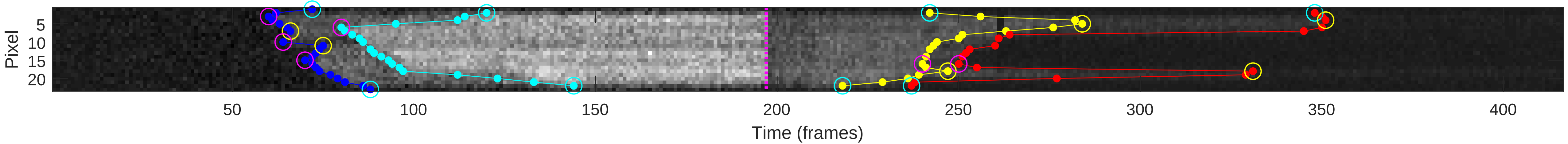

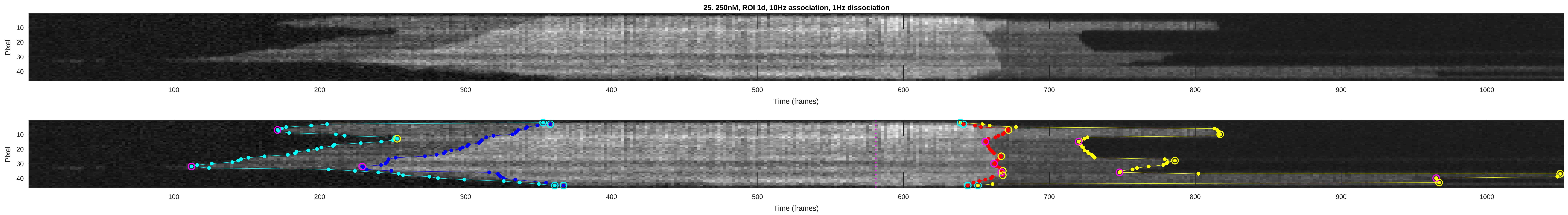
25. 250nM, ROI 1d, 10Hz association, 1Hz dissociation
Pixel
10
20
30
40
100
200
300
400
500
600
700
800
900
1000
Time (frames)
Pixel
10
20
30
40
100
200
300
400
500
600
700
800
900
1000
Time (frames)

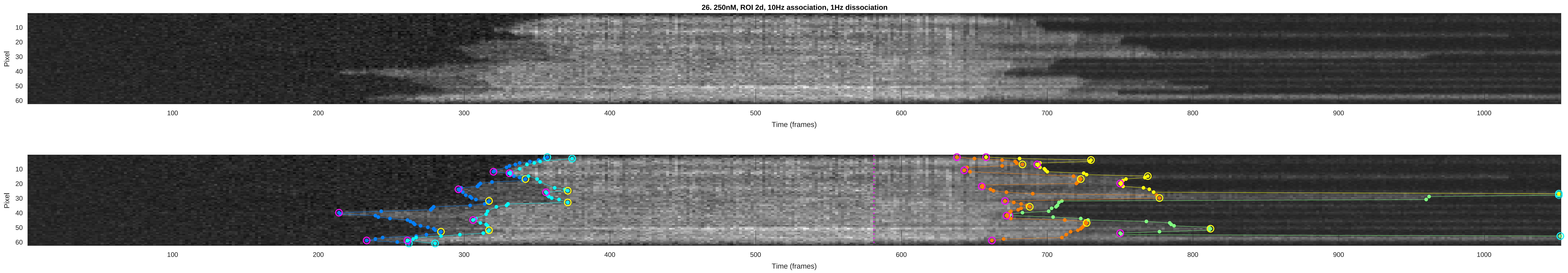
26. 250nM, ROI 2d, 10Hz association, 1Hz dissociation
Pixel
10
20
30
40
50
60
100
200
300
400
500
600
700
800
900
1000
Time (frames)
Pixel
10
20
30
40
50
60
100
200
300
400
500
600
700
800
900
1000
Time (frames)

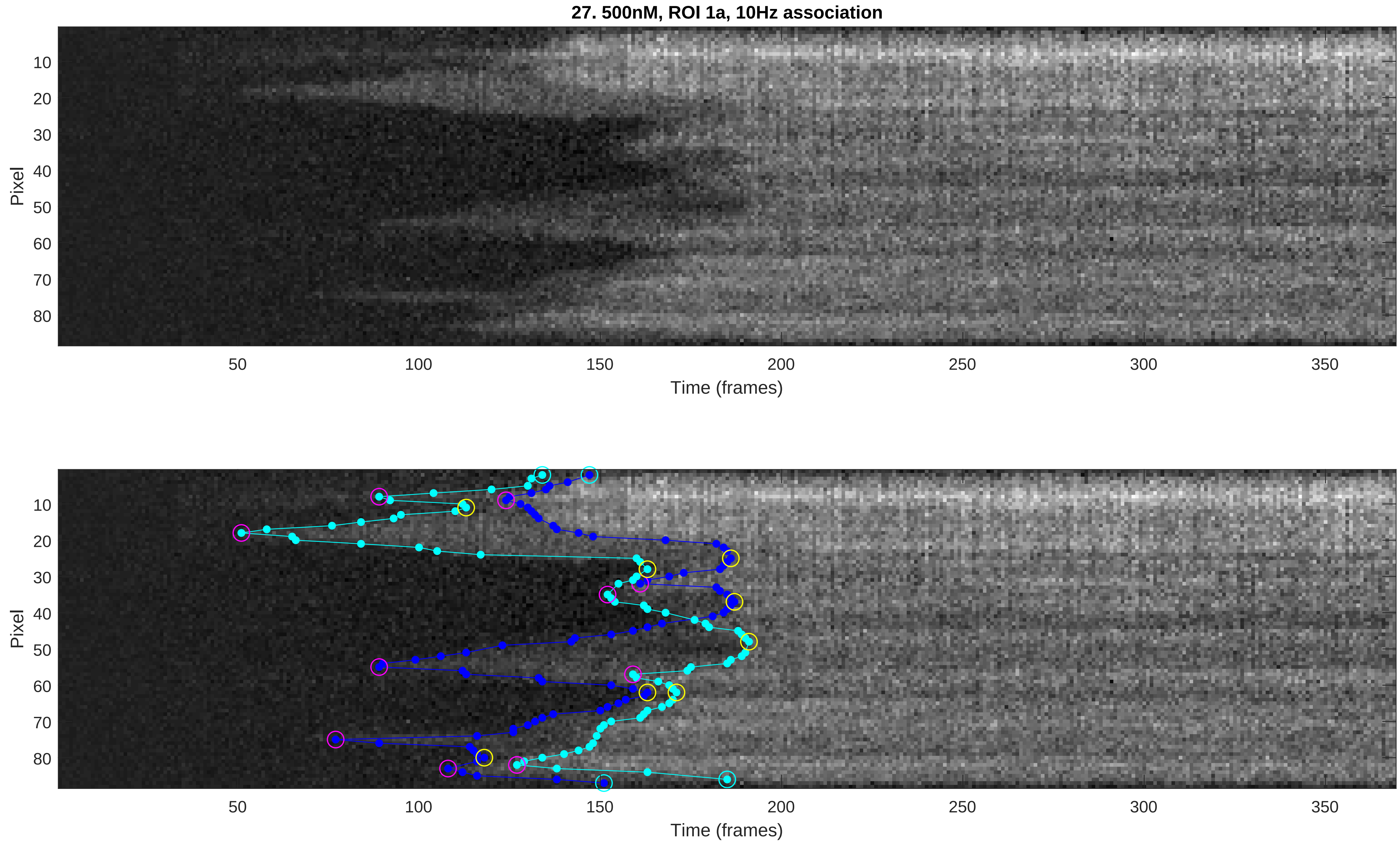
27. 500nM, ROI 1a, 10Hz association
Pixel
10
20
30
40
50
60
70
80
50
100
150
200
250
300
350
Time (frames)
Pixel
10
20
30
40
50
60
70
80
50
100
150
200
250
300
350
Time (frames)

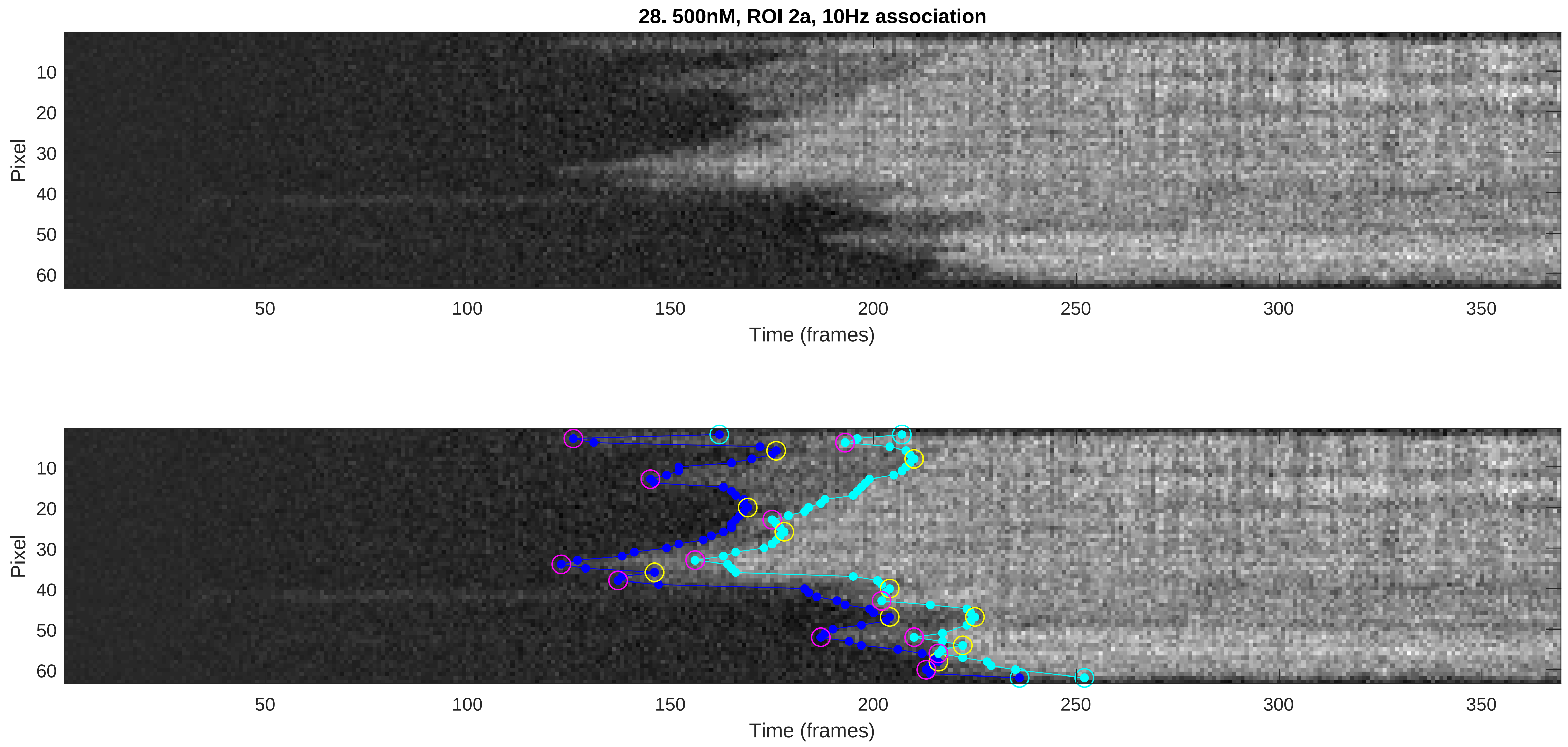

28. 500nM, ROI 2a, 10Hz association
Pixel
10
20
30
40
50
60
50
100
150
200
250
300
350
Time (frames)
Pixel
10
20
30
40
50
60
50
100
150
200
250
300
350
Time (frames)

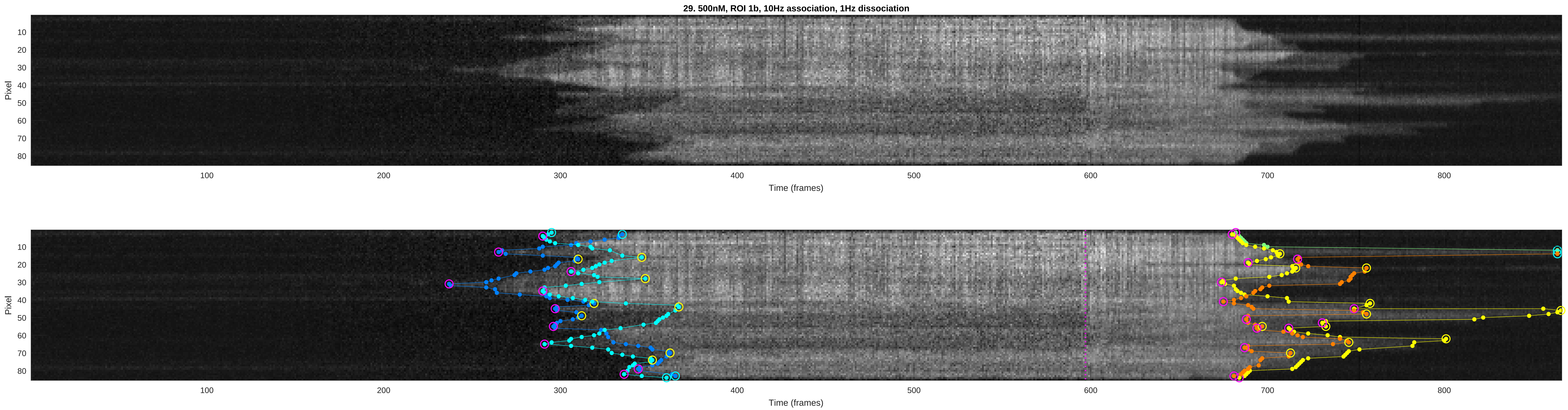
29. 500nM, ROI 1b, 10Hz association, 1Hz dissociation
Pixel
Time (frames)
Pixel
Time (frames)

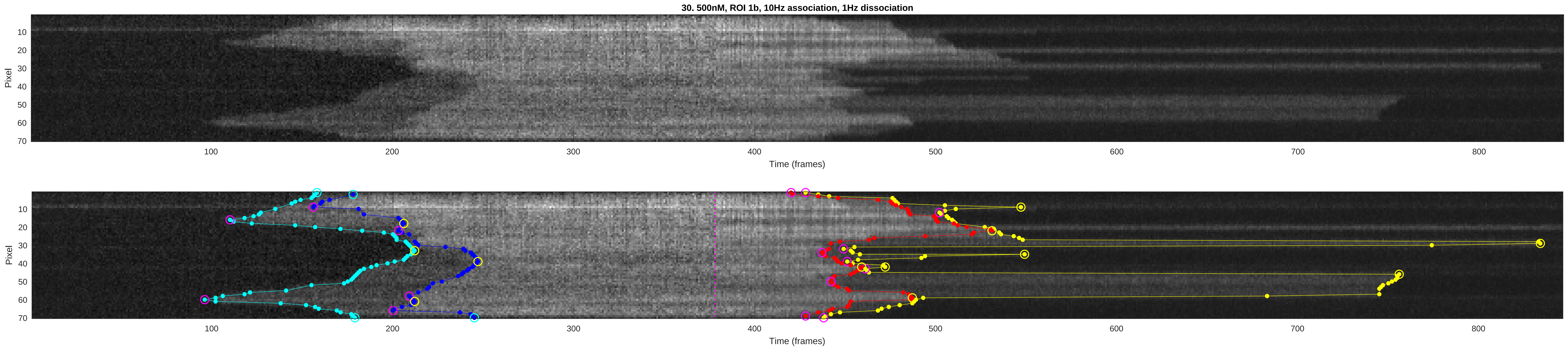
30. 500nM, ROI 1b, 10Hz association, 1Hz dissociation
Pixel
Time (frames)
10
20
30
40
50
60
70
100
200
300
400
500
600
700
800
Pixel
Time (frames)
